# Relatively slow stochastic gene-state switching in the presence of positive feedback significantly broadens the region of bimodality through stabilizing the uninduced phenotypic state

**DOI:** 10.1371/journal.pcbi.1006051

**Published:** 2018-03-12

**Authors:** Hao Ge, Pingping Wu, Hong Qian, Xiaoliang Sunney Xie

**Affiliations:** 1 Biodynamic Optical Imaging Center (BIOPIC), Peking University, Beijing, P.R.China; 2 Beijing International Center for Mathematical Research (BICMR), Peking University, Beijing, P.R.China; 3 School of Mathematical Sciences and Centre for Computational Systems Biology, Fudan University, Shanghai, P.R.China; 4 Department of Applied Mathematics, University of Washington, Seattle, Washington, United States of America; 5 Department of Chemistry and Chemical Biology, Harvard University, Cambridge, Massachusetts, United States of America; University of Michigan, UNITED STATES

## Abstract

Within an isogenic population, even in the same extracellular environment, individual cells can exhibit various phenotypic states. The exact role of stochastic gene-state switching regulating the transition among these phenotypic states in a single cell is not fully understood, especially in the presence of positive feedback. Recent high-precision single-cell measurements showed that, at least in bacteria, switching in gene states is slow relative to the typical rates of active transcription and translation. Hence using the *lac* operon as an archetype, in such a region of operon-state switching, we present a fluctuating-rate model for this classical gene regulation module, incorporating the more realistic operon-state switching mechanism that was recently elucidated. We found that the positive feedback mechanism induces bistability (referred to as deterministic bistability), and that the parameter range for its occurrence is significantly broadened by stochastic operon-state switching. We further show that in the absence of positive feedback, operon-state switching must be extremely slow to trigger bistability by itself. However, in the presence of positive feedback, which stabilizes the induced state, the relatively slow operon-state switching kinetics within the physiological region are sufficient to stabilize the uninduced state, together generating a broadened parameter region of bistability (referred to as stochastic bistability). We illustrate the opposite phenotype-transition rate dependence upon the operon-state switching rates in the two types of bistability, with the aid of a recently proposed rate formula for fluctuating-rate models. The rate formula also predicts a maximal transition rate in the intermediate region of operon-state switching, which is validated by numerical simulations in our model. Overall, our findings suggest a biological function of transcriptional “variations” among genetically identical cells, for the emergence of bistability and transition between phenotypic states.

## Author summary

Identifying the mechanism underlying the coexistence of multiple stable phenotypic states has been a challenging scientific problem for more than half a century, and an appropriate mathematical model at the single-cell level is also in high demand. Single-cell measurements conducted in the past ten years have shown that gene-state switching is slow relative to the typical rates of active transcription and translation; hence the recently proposed fluctuating-rate model is a good candidate for describing the single-cell dynamics. We use the classic gene regulation module of the *lac* operon as an archetype and build a specific fluctuating-rate model based on the recently identified operon-state switching mechanism. This model is analyzed to dissect the interplay between positive feedback and the stochastic switching of gene states in the emergence of bistability/multistablity and the transition between phenotypic states. We show that relatively slow operon-state switching stabilizes the uninduced state and that the positive feedback stabilizes the induced state. Thus, the parameter range for bistability is significantly broadened. In addition, recently proposed landscape theory and rate formula predict opposite phenotype-transition rate dependence on operon-state switching rates for the two types of bistability.

## Introduction

Individual cells of a given genotype can exhibit various phenotypes. The phenotype of a cell usually refers to distinct characteristics (static and dynamic, physical or chemical) and the associative biological functions of the cell. Extending the central dogma of molecular biology, it is now accepted that the behavior of a single cell is determined by both the genomic polynucleic acid sequence and the dynamics of intracellular biochemical networks in space and time. The biochemical reactions inside cells serve as the immediate environment for the genome, where genotypic information resides. It is only through intracellular biochemistry that extracellular conditions can interact with genes.

Based on this perspective, we propose the following: for a population of cells with identical genomes and extracellular conditions, each phenotype can be represented by a cluster of single-cell data defined as a peak (e.g., modal value) in the multi-dimensional histogram of biomolecular copy-numbers measured at steady state. In general, the peak is a sizable region in the vast biochemical kinetic space, which is known as an attractor in chemical kinetics [1, 2]. Multiple peaks naturally discretize the space; at a given instance in time, a single cell can reside in one of these discrete states.

More interestingly, a homogeneous cell population responds to a varying environment through changes in the distribution among discrete phenotypic states, rather than through gradual adaptation to an intermediate state [3]. At a single-cell level, this observation is known as all-or-none [4]. Furthermore, it has recently been shown that a steady-state multi-modal distribution can be recovered after a subpopulation of cells under a peak is removed [5, 6], indicating that dynamic interconversion between phenotypic states occurs within a single cell. In a sufficiently long time, each single cell is considered ergodic among the different phenotypes. The coexistence of multiple phenotypic states diversifies clonal cells; and provides a non-genetic evolutionary advantage for survival in unpredictable environments [7–9].

Recent experiments have revealed that the dynamics of a single cell are essentially stochastic, as there is only a single copy of DNA inside a typical cell, which leads to stochastic mRNA and protein production [10, 11]. Both transcriptional and translational events have been shown to occur in stochastic bursts [10, 12–20] indicating that the gene state switches stochastically and is relatively slow compared with the typical rates of active transcription and translation. The stochastic operon-state switching of the *lac* operon has also been shown to be crucial for the change in a cell’s phenotype [3, 13], which highlights the importance of single-molecule events inside the cell.

In terms of quantitative biochemical kinetics, the temporal evolution of the probability distribution of a well-mixed reaction system is governed by a Chemical Master Equation (CME) [21], from which the corresponding stochastic trajectory of a single cell can be computationally simulated. We have recently shown, using a toy model of gene regulation, that when the rate of gene-state switching is low relative to the typical rates of active transcription and translation, the full CME can be reduced to a single-molecule fluctuating-rate model, in which the dynamics of mRNA and protein copy numbers at each given gene state follow deterministic dynamics while transcription rates fluctuate due to stochastic gene-state switching [22], which is necessary for spontaneous phenotypic state transitions.

In the full Chemical Master Equation, the copy-number fluctuations of mRNA and protein resulting from stochastic synthesis and degradation are present, which prevent us from studying the role of only the stochastic gene-state switching. However, in fluctuating-rate models, stochastic gene-state switching is the only source of randomness, the conclusions drawn from which are much more clean and unambiguous. On the other hand, although numerical simulations of full Chemical Master Equation can be practical, theoretical analysis is still difficult to implement; while solid theoretical foundations have already been proposed for fluctuating-rate models, which are also called piecewise deterministic Markov processes [22, 23]. The fluctuating-rate model is easier to implement both theoretically and numerically. Therefore, it is a good candidate for studying single-cell dynamics, especially towards investigating the role of only the stochastic gene-state switching.

So far, the exact role of stochastic gene-state switching that occurs during the transition between phenotypic states in a single cell is unclear, especially in the presence of positive feedback. In the present study, we address this problem using the *lac* operon as an archetype. Recent experiments have shown that the switching of operon states of the *lac* operon is slow compared with typical rates of active transcription and translation. Thus, in such a region of operon-state switching, we propose to explore the single-molecule fluctuating-rate model in quantitative detail, by incorporating the previous described operon-state switching mechanism [3]. This mathematical model illustrates the emergence of discrete phenotypic states from detailed nonlinear biochemical kinetics, and the robustness of such cellular states follows naturally. Although in general, positive feedback is necessary for bistability in a biochemical network, we show that the stochasticity in operon-state switching of an individual cell is able to not only trigger stochastic transitions between phenotypic states, but also significantly broaden the range of environmental parameters under which bistability occurs. The bistability that occurs in the absence of stochasticity is called deterministic bistability, while the bistability which occurs in the presence of stochasticity but beyond the parameter range of deterministic bistability is called stochastic bistability.

We further show that stochastic operon-state switching must be extremely slow to trigger stochastic bistability (bimodal distribution) by itself in the absence of positive feedback. On the other hand, positive feedback is known to be able to maintain a stable state [24–26], hence with the help of positive feedback, the induced state is stabilized beyond the range of deterministic bistability, even when the rates of stochastic operon-state switching is only within the physiological region. However, positive feedback is not able to stabilize the uninduced state within the same parameter region. We show that the uninduced state is instead stabilized by the relatively slow operon-state switching. Together, the mechanism of significantly broadened parameter range of bistability is explained. We further predict how the phenotype-transition rates vary with operon-state switching rates under each type of bistability. We also illustrate that the maximal transition rates between different phenotypic states are achieved with an intermediate rate of operon-state switching, which is a phenomenon that was predicted previously [27] and is explained using a recently proposed phenotype-transition rate formula.

Finally but not the least, we not only explained the previously reported experimental discoveries in the present study, but also further refined some earlier conclusions that were not as precisely presented, such as the effect of DNA looping as well as the concept and quantification of thresholds of phenotype transitions.

## Results

The *lac* operon of *E. coli*, which involves multiple transcription factors, was the first complex model of gene regulation to be elucidated. It consists of a promoter, a terminator, an operator and three adjacent structural genes (*lacZ*, *lacY*, and *lacA*). *lacZ* encodes *β*-galactosidase, an intracellular enzyme that catalyzes the transformation of the disaccharide lactose to glucose and galactose, while *lacY* encodes *β*-galactoside permease, a membrane-bound transport protein that transports extracellular lactose into the cell. The *lac* operon remains inactive when there is no extracellular lactose available, or if there is a more readily-available energy source, such as glucose. However, it is rapidly activated when lactose is present(in the absence of glucose), due to positive feedback.

**Fig 1:**
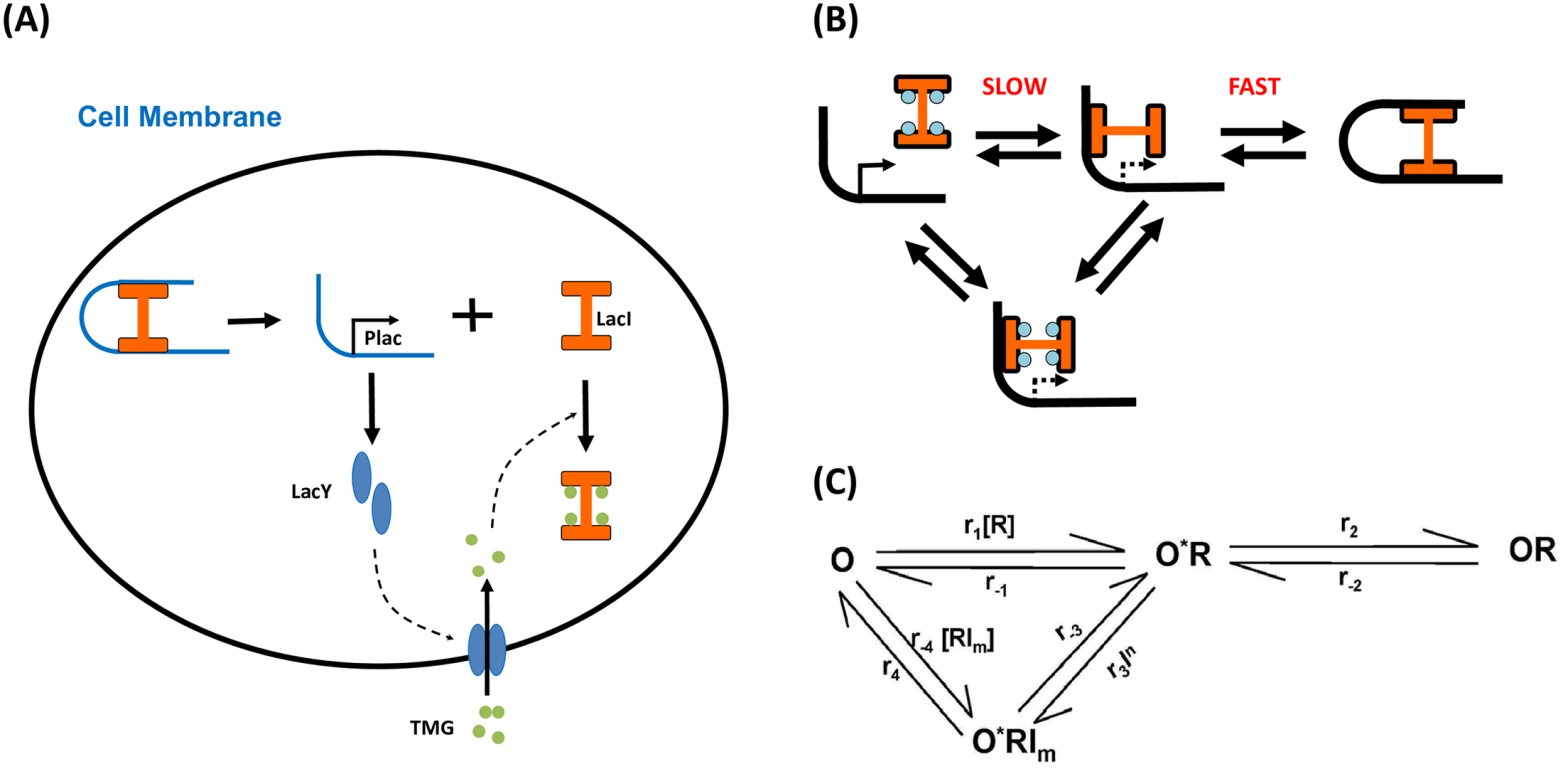
Overview of the model. (A) Regulatory mechanism of the *lac* operon. Expression of permease increases the intracellular concentration of the inducer TMG (thiomethyl *β*-D-galactoside), which removes the repressor LacI from the promoter, leading to increased expression of permease. Hence the repressor LacI and permease LacY form a positive feedback loop. (B) Cartoon showing the dynamics of operon states. (C) Diagram of the Markovian jumping process of operon states. The *O* state denotes the free operon; the O*R state denotes the operon bound to the repressor at the auxiliary *lac* operator *O*_2_ or *O*_3_(partial dissociation); the *OR* state denotes the repressor bound to the operon at both the major and auxiliary *lac* operators; the O*RI_*m*_ state denotes the repressor bound to the operon at the auxiliary *lac* operator *O*_2_ or *O*_3_ and to the inducer.

In the absence of lactose, the production of *β*-galactosidase is inhibited: an intracellular regulatory protein known as the lactose repressor (*lacI* gene product) binds to the *lac* operator. In the presence of lactose, the repressor’s affinity for the *lac* operator is decreased by allolactose, whose production from lactose is catalyzed by *β*-galactoside. As the repressor’s affinity decreases, RNA polymerase transcribes the *lac* genes, leading to a high level of the encoded proteins and consequent digestion of more lactose (Fig 1A). In wet-lab experiments, inducers such as the lactose analog thiomethyl *β*-D-galactoside (TMG) are used instead of lactose, because such inducers are not readily digested and therefore remain at a constant concentration.

The *lac* repressor molecule is a tetramer of identical subunits. Under repressed conditions, one dimer binds to the major *lac* operator *O*_1_, and a second dimer binds to one of the weaker auxiliary operators, *O*_2_ or *O*_3_, together forming a DNA loop. Choi et al. [3] have investigated the molecular mechanism of the transition of *E. coli* cells from one phenotype to another via *lac* operon. At intermediate inducer concentrations, a population of genetically identical cells will exhibit two distinct phenotypes: fully induced cells and uninduced cells. Choi et al. observed a basal level of expression in uninduced cells as a result of the partial dissociation of the tetrameric repressor from the operator *O*_1_ on looped DNA. In contrast, the rare occurrence of complete dissociation of the repressor results in large bursts of permease expression, which trigger the induction of the *lac* operon. Therefore, stochastic single-molecule switching between operon states is responsible for the change in the phenotype of the cell.

### Single-molecule fluctuating-rate model with recently identified operon-state switching mechanism

Stochastic gene-state switching is a major source of stochasticity inside a single cell [10, 13, 28], and even responsible for the phenotype transition [3]. By evoking the recently identified stochastic gene-state switching mechanism, we propose a single-molecule fluctuating-rate model for the lactose operon, introducing the fluctuating transcription rates into the deterministic dynamics described in previous studies [4, 29, 30].

The deterministic dynamics of all other chemical species under each operon state in the fluctuating-rate model consist of several differential equations representing the temporal evolution of mRNA, LacY polypeptides, and the intracellular inducer concentrations. The stochastic kinetics of the operon states are described in Fig 1B and 1C, and are modeled by a simple Markovian jumping process. The state *O* denotes the free operon; the state O*R denotes the operon with the repressor bound only at the auxiliary *lac* operator(partial dissociation); the state *OR* denotes the operon with the repressor bound at both the major and auxiliary *lac* operators; and the state O*RI_*m*_ denotes the operon bound by both the repressor at the auxiliary *lac* operator and inducer molecules. While the inducer is unlikely to interact with the fully bound tetrameric repressor, it could conceivably bind to the inducer once a dimer head of the repressor dissociates.

Traditionally, the O*RI_*m*_ complex, which contains both the repressor and the inducers bound on the operon, is omitted in mathematical models. Such an over-simplified model cannot explain why the repressor binds stably to DNA in the absence of inducer, and is released rapidly in the presence of inducer. Previous models, assuming either *O* + *R* ⇋ *OR* or *O* + *R* ⇋ O*R ⇋ *OR*, imply that the rate of the complete dissociation of the repressor is independent of the intracellular inducer concentration. However, data show that when the intracellular inducer concentration is high, the frequency of complete dissociation can also be high (0.01 minute^−1^) (Fig 2A in [3]). Alternatively, when the intracellular inducer concentration is low, the frequency of complete dissociation events is low and shows very weak concentration dependence (Fig 3D in [3]). Therefore it appears that the repressor also binds the inducer when bound to the operon.

In addition, the repressor has 2 different binding constants (i.e. *K* and 1/*K*_3_, see S1 Text for details) for the inducer, depending on whether the repressor is already bound to DNA [31, 32], which are 10 to 100-fold apart. Accordingly, when inducer concentrations are below the lower binding constant, there is weak concentration dependence of the complete dissociation rate, whereas once the inducer concentration approaches the higher binding constant (100 *μ*M), the complete dissociation rate increases dramatically via the *OR* → O*R → O*RI_*m*_ → *O* pathway shown in Fig 1B and 1C. This is the basic type of *lac* operon induction with which most molecular biologists are familiar. However, the role of single-molecule fluctuations of DNA transcription under intermediate concentrations of inducer was unclear prior to the work of Choi, et al. [3].

The parameters of our model were obtained either directly from experimental measurements or through fitting the predictions of the model to experimental data, as explained in S1 Text.

### Relatively slow gene-state switching induces stochastic bistability with a much broadened parameter range

#### Positive feedback induces deterministic bistability

When stochastic operon-state switching is very rapid, the dynamics of a single cell are well-described by a deterministic mean-field equation (See Eq 3). Theoretical chemists refer to this as the adiabatic limit [27, 33]. Such systems exhibit bistability over a certain range of environmental parameters (i.e., co-existence of two phenotypic states), as long as the synthesized gene product positively regulates its own synthesis in a sigmoidal fashion [34]. Positive feedback increases protein levels, which leads to a higher rate of synthesis and an even greater protein levels. However, positive feedback also implies lower protein levels resulting in reduced synthesis and a further decrease in protein levels (with the presence of degradation). Therefore, there must exist a critical protein level (threshold) in a single cell above which protein levels increase until complete saturation (on-state or induced state) is reached and below which protein levels drop until nearly reaching zero (off-state or uninduced state). Hence, there exist three steady states in the presence of strong positive feedback: two stable states that are separated by an unstable state, which is referred to as the “threshold” (or saddle point, similar to a transition state in molecular biophysics). When a system deviates from the unstable threshold, the deviation becomes even greater due to positive feedback until the system reaches a stable steady state. This mechanism is referred to as deterministic bistability, in contrast to the case below, which is caused by stochastic fluctuations without a deterministic counterpart.

**Fig 2:**
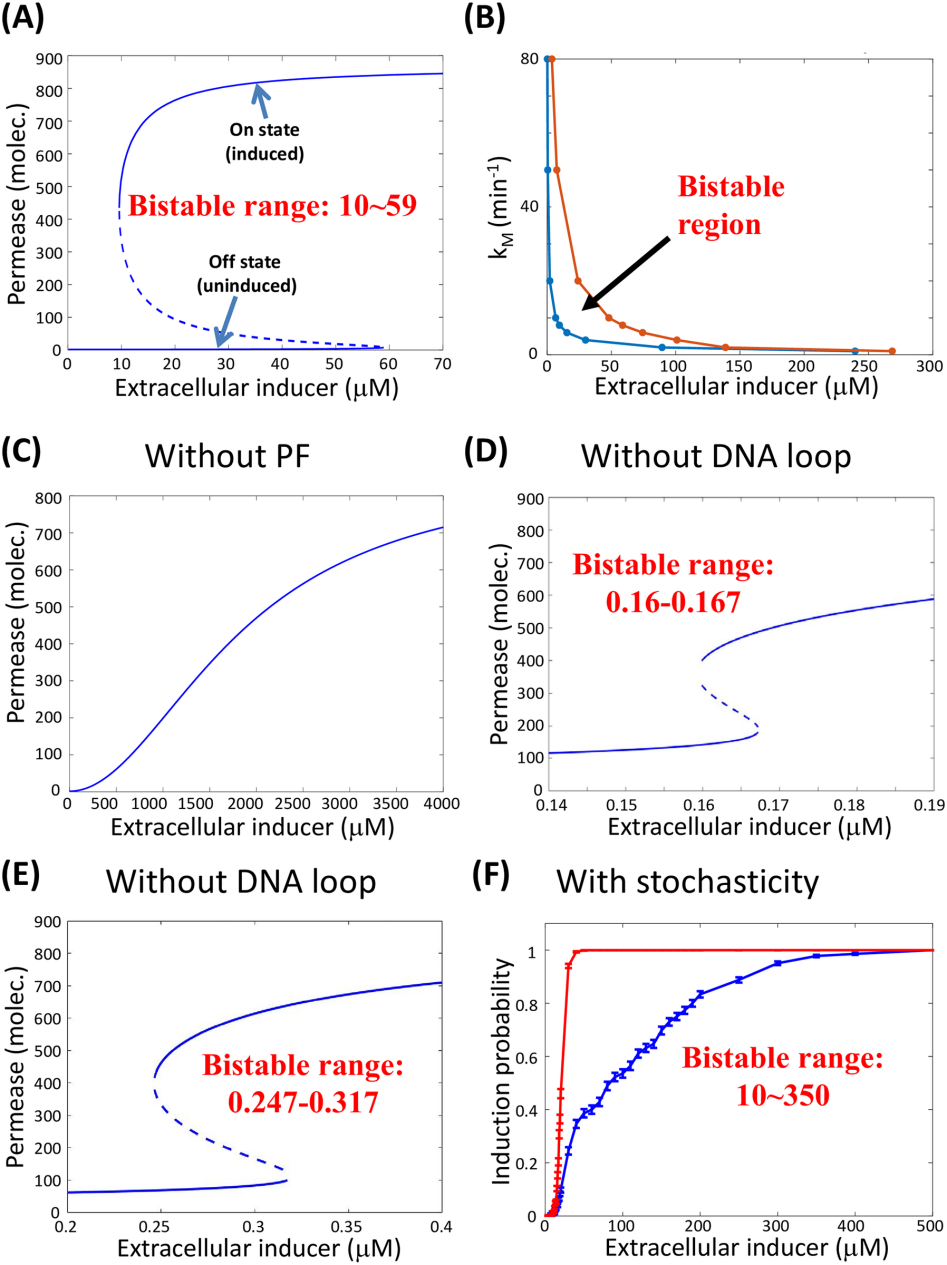
Bistability with and without stochastic operon-state switching. (A) Deterministic bifurcation diagram for wild-type cells. There are two saddle-node bifurcations occurring around *I*_*e*_ = 10*μ*M and 59*μ*M. (B) Deterministic bifurcation diagram containing both the active transcriptional rate *k*_*M*_ and the extracellular inducer concentration *I*_*e*_. The wild-type cells exhibit deterministic bistability inside the parameter region between the blue and brown lines and exhibit monostability otherwise. (C) Deterministic bifurcation diagram of the mutant cells without positive feedback. (D)(E) Deterministic bifurcation diagrams with different association constants for the repressor bound to the operon in the absence of a DNA loop: 5 *molec.*^−1^ (D), 8 *molec.*^−1^ (E). (F) Stochastic hysteresis response of the probability of induction for wild-type cells. Initial conditions: uninduced (blue line) or fully induced (red line) cells with a period of *T* = 2000 min. The extracellular inducer concentration must exceed over 350*μ*M to completely activate initially uninduced cells, whereas it must decrease below 10*μ*M to completely deactivate the initially induced cells. See S1 Text for parameter values.

In addition to the all-or-none bistable system, there is another common decision-making mechanism in cells: the ultrasensitive system with a graded response. These mechanisms are not incompatible with each other, and cells have the ability to convert one to the other and vise versa [4]. Hence, a bifurcation diagram can be utilized to precisely represent the complete range of environmental parameters over which the system is bistable. Fig 2A shows the steady-state copy number of permease as a function of the extracellular concentration, *I*_*e*_, of inducer in the wild-type *lac* operon in the presence of positive feedback. The bifurcation diagram is always accompanied by a hysteresis loop [34, 35]. In the absence of intrinsic stochasticity, when the environmental parameter increases, the system remains in the off-state until it is no longer stable. Similarly, when the parameter decreases but remains within the bistable region, the on-state remains stable, although the off-state reappears. Hysteresis protects the bistable system from repeatedly transiting back and forth between the two phenotypic states when the environmental parameter is near one of the critical values at which the bifurcations occur. Phenotype transitions involving hysteresis are driven by slow external modulation, while spontaneous transitions under fixed parameters require randomness.

In addition to the extracellular inducer concentration, *I*_*e*_, other parameters of the system can also be tuned experimentally. For instance, an increase or decrease in the maximum transcriptional rate can be achieved by increasing the number of operons in the cell or changing the concentrations of other transcription factors. We computed the deterministic bifurcation diagram (bistable or monostable) with both the active transcriptional rate, *k*_*M*_, and the extracellular inducer concentration, *I*_*e*_ (Fig 2B). The system is bistable inside the parameter region between the blue and brown lines and is monostable otherwise; it is an analog of a first-order phase transition [36]. We show that the bistable range of extracellular inducer concentrations becomes increasingly narrow and then disappears when *k*_*M*_ either increases or decreases from the value *k*_*M*_ = 8min^−1^ for wild-type cells, which is known as the cusp phenomenon [37].

In mutant strains that cannot transport lactose or the inducer into the cell, positive feedback is disrupted. In this case, there is only one steady state (see Eq 3), which implies that positive feedback is necessary for deterministic bistability (Fig 2C). Experiments have also indicated that wild-type cells do not exhibit bistability without forming DNA loops as the repressor bound to the operon [3]. However, our model demonstrates that bistability still exists without DNA loops, although the range of bistability becomes much narrower and is therefore harder to detect (Fig 2D and 2E).

#### Relatively slow operon state switching broadens the parameter range of bistability in the presence of positive feedback

Spontaneous transitions between the on-state and the off-state occur in a single cell. In mathematical models, stochasticity causes spontaneous transitions in a deterministic bistable system and might also cause systems without deterministic bistability to exhibit bistable phenomena, i.e., a bimodal distribution. To determine the parameter range for bistability in a stochastic system, we considered entire populations of cells starting from either the on-state or the off-state at time zero and determined the fraction of cells in the off state under different extracellular inducer concentrations after a certain amount of time (Fig 2F, also see Materials and methods). This behavior is referred to as hysteresis and can be directly measured in single-cell experiments [4]. In wild-type cells, we found that the range of hysteresis was much wider than in the deterministic bistable system shown in Fig 2A. We then directly simulated the copy-number distribution of permease, which confirmed the broadened range of bistability (S2 Fig). The two induction curves presented in Fig 2F would merge and the same fraction of phenotypic states would be achieved regardless of the different initial states at which the cells start, only if the extracellular inducer concentration remains constant for an extremely long period of time. For cellular phenotypes, this “extremely” long period of time can be on the order of months or years, which completely beyond the relevant time scale for cell division and typical experiments. This period is the origin of the ambiguity concerning the threshold in the presence of stochasticity (see below).

Furthermore, a cusp phenomenon similar to that shown in Fig 2B still exists in the presence of intrinsic stochasticity, when the *k*_*M*_ and *I*_*e*_ are considered. Bistability becomes more indistinct when *k*_*M*_ becomes either smaller or larger (S4 and S5 Figs). Therefore, the wild-type value of *k*_*M*_ is somewhat optimized. This phenomenon gives cells the opportunity to transition between a hysteresis response system and an ultrasensitive graded response system. The cusp phenomenon could be examined by replacing *k*_*M*_ with other parameters in the system.

#### Stochastic bistability in the absence of positive feedback requires extremely slow operon-state switching

It is known that stochasticity can induce bistability that has no macroscopic counterpart and such a noise-induced bistability, called stochastic bistability, arises from slow gene-state switching [33, 38–43]. Theoretical chemists refer to this scenario as “non-adiabatic”, which is analogous to slow-moving nuclei in quantum mechanical atoms, which is also similar to the quasi-static regime of enzyme kinetics [44]. This phenomenon is purely stochastic; i.e., there is no determinstic bistability in the mean-field model that describes in vitro biochemical experiments with large amounts of purified chemicals involving the same parameters.

However, in the absence of positive feedback, with the same parameters of wild-type cells, we found that the stationary distribution was broad and did not exhibit distinct bistability (S3 Fig). This result implies that the operon-state switching inside a wild-type cell is not sufficiently slow to exhibit bistability without positive feedback.

**Fig 3:**
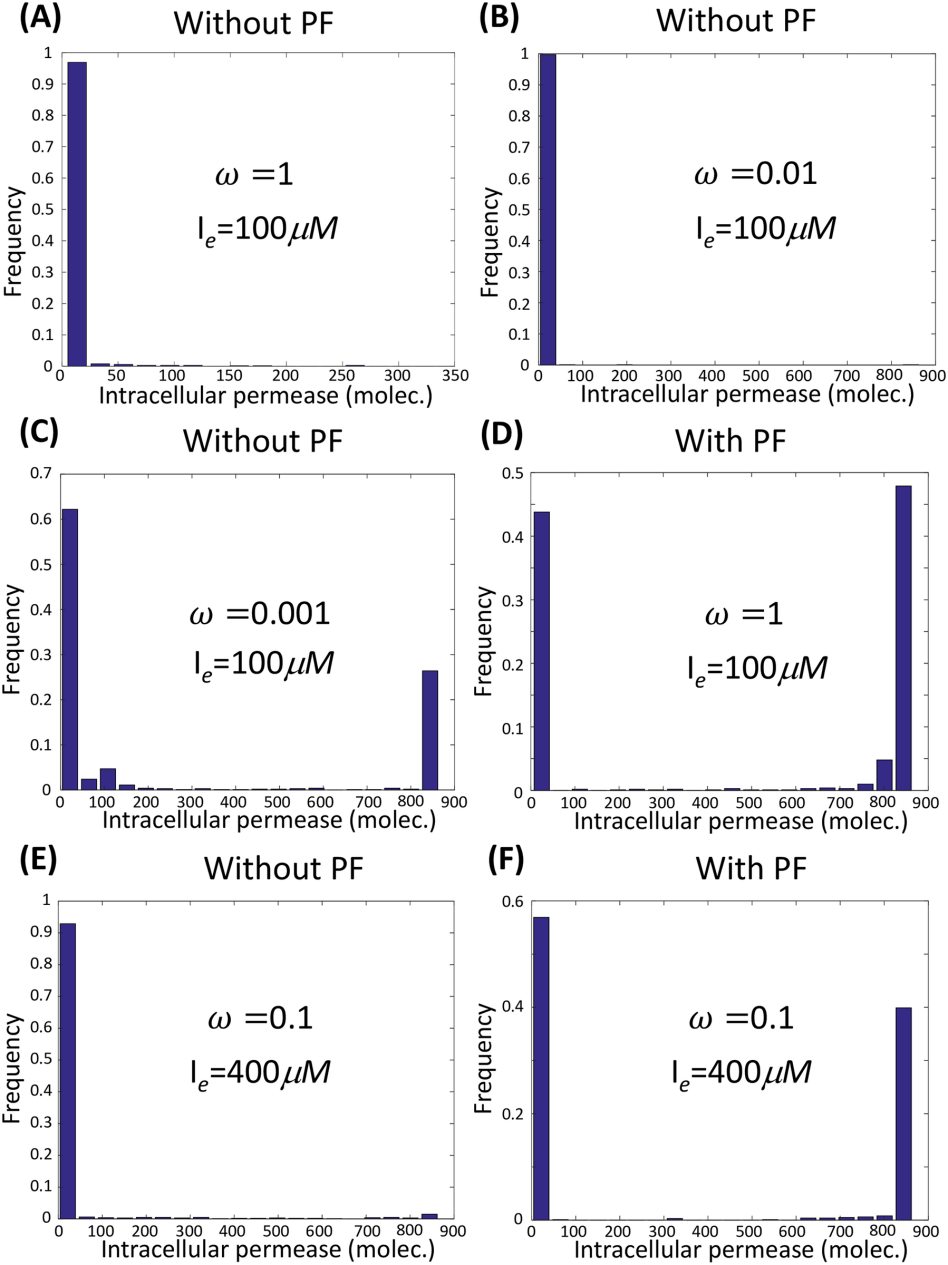
Positive feedback stabilizes the induced state. (A-C) Extremely slow operon-state switching is necessary to induce purely stochastic bistability without positive feedback. (D-F) In the presence of positive feedback, the induced state is stabilized, and a bimodal distribution emerges, even when operon-state switching rates are within the physiological region.

We introduce the dimensionless parameter *ω* (See Material and methods), which characterizes the rate of switching among multiple gene states. Mathematically, *ω* can be defined as the ratio of switching rates among different gene states with respect to the wild-type rates or the protein decay rate. We choose the former, in which *ω* = 1 corresponds to the wild-type rates. Further simulation showed that the switching rates among operon states must be at least 100-1000 times slower than in the wild-type cells (*ω* = 0.01 − 0.001) in order to trigger purely stochastic bistability in the absence of positive feedback (Fig 3A–3C), which is rarely possible.

#### Positive feedback and slow operon-state switching stabilize the induced and uninduced states respectively

In the presence of positive feedback, it is beneficial to stabilize the induced state as long as the extracellular inducer concentration is not too low, even when the switching rates among operon states are within physiological regions (comparing Fig 3A and 3E with Fig 3D and 3F). In the induced state, the repressor is always fully dissociated from the operon; once the operon is repressed, positive feedback keeps the intracellular inducer concentration at a relatively high level; therefore, the repressor protein is forced to fully dissociate from the operon rapidly, which stabilizes the induced state.

**Fig 4:**
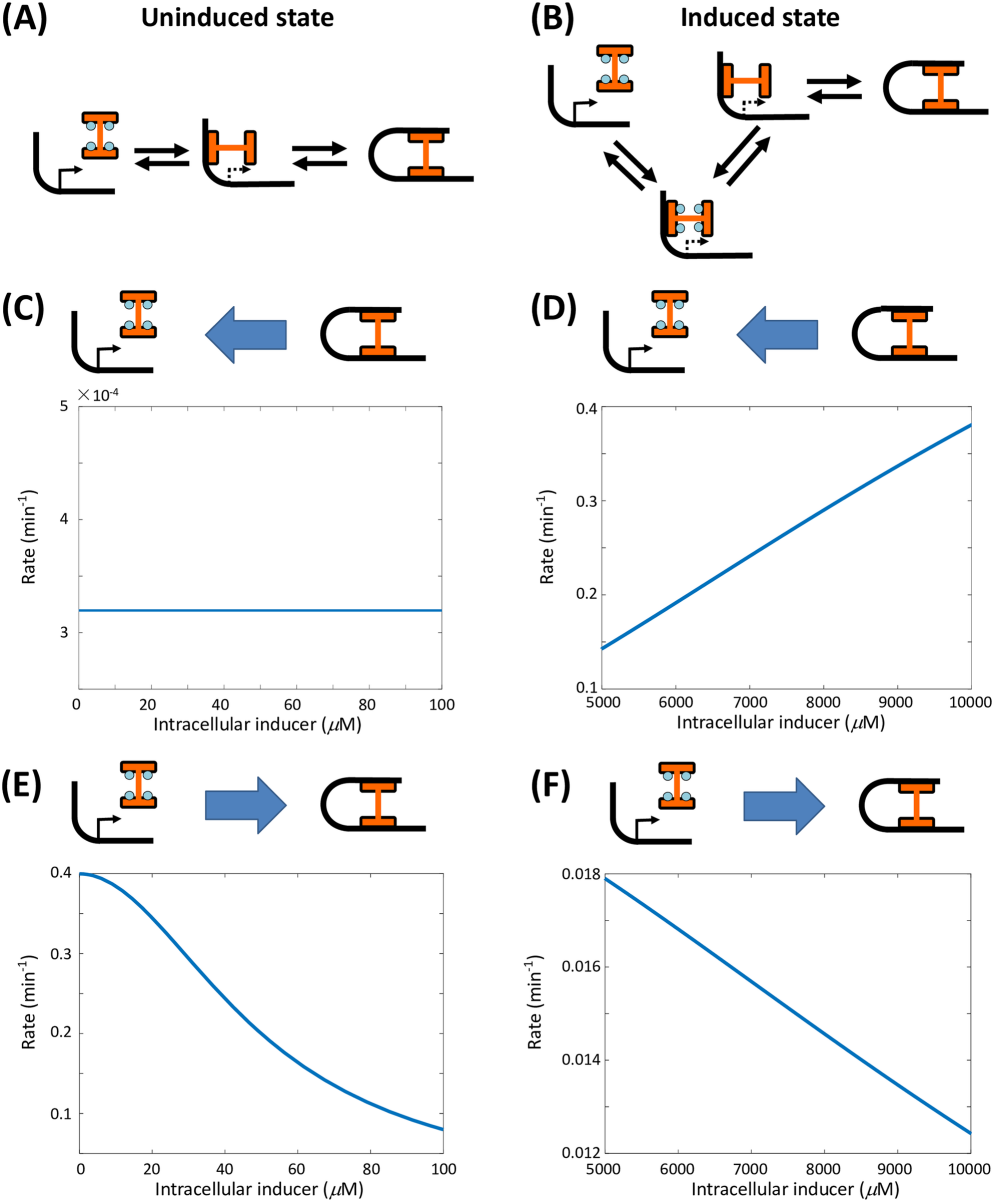
Major transit pathways and transition rates between fully repressed and fully dissociated operon states. (A, B) The major transit pathways between fully repressed and fully dissociated operon states in the uninduced and induced phenotypic states. (C-F) Transition rates between fully repressed and fully dissociated operon states in the uninduced and induced phenotypic states with very low and high intracellular inducer concentrations respectively. The transition rates from the fully repressed operon state to the fully dissociated state in the uninduced phenotypic state are the lowest, which stabilizes the uninduced state, even outside of the parameter range of deterministic bistability.

We calculated the mean transition time between the fully repressed operon state and the fully dissociated operon state of wild-type cells, under an intracellular inducer concentration that is very high (induced state) or quite low (uninduced state) (Fig 4). The main pathways underlying the induced state and the uninduced state are different (Fig 4A and 4B). Under the uninduced state, the mean transition time (the reciprocal of rate) from the fully repressed operon state to the fully dissociated operon state is quite long (Fig 4C), which stabilizes the uninduced state, and the mean transition time backwards is much shorter (Fig 4E). On the contrary, the mean transition time back and forth between the fully repressed operon state and fully dissociated state in the induced state is also much shorter, which implies that the stability of the induced state can not be guaranteed by the stochastic switching between different operon states.

As the strength of stochasticity decreases, i.e. increasing the rates for stochastic switching among operon states, the broadened parameter range for bistability in Fig 2F becomes narrower and narrower, approaching the deterministic limit in Fig 2A (See S12 Fig). It is because the rapid stochastic switching among operon states is not able to stabilize the uninduced state any more outside the range of deterministic bistability. We also have tuned the strength of positive feedback, i.e. the parameter *K*, which is the equilibrium constant of the binding reaction between the repressor and inducer. As the strength of positive feedback decreases, i.e. increasing the parameter *K*, the capability of stabilizing the induced state also decreases (See S14 Fig).

Together, positive feedback and stochastic operon-state switching significantly broaden the parameter range of bistability. When the extracellular inducer concentration is low, positive feedback cannot stabilize the induced state, whereas when the extracellular inducer concentration is high, positive feedback and slow fluctuations of operon states stabilize the induced and uninduced states, respectively. This result explains why the broadened parameter range of bistability can only be visualized on the right-hand-side of the deterministic bifurcation diagram in Fig 2A.

### Stochastic transition between different phenotypic states

In addition to the mechanism of the emergence of bistability, we also sought to quantitatively investigate the transition rates between different phenotypic states and the more detailed molecular mechanisms that trigger them.

#### How a single-molecule event determines the phenotype of a wild-type cell

There are two kinds of bursts in the *lac* operon system: small bursts due to partial dissociation and large bursts due to complete dissociation of the repressor from the operon. We confirmed previously reported experimental observations [3] (using the mathematical interpretations in the S1 Text and see S6 Fig): the size and frequency of small bursts are nearly independent of the intracellular inducer concentration; and for large bursts, in the absence of positive feedback, size always increases with the intracellular inducer concentration, while the frequency is invariant under a low inducer concentration.

**Fig 5:**
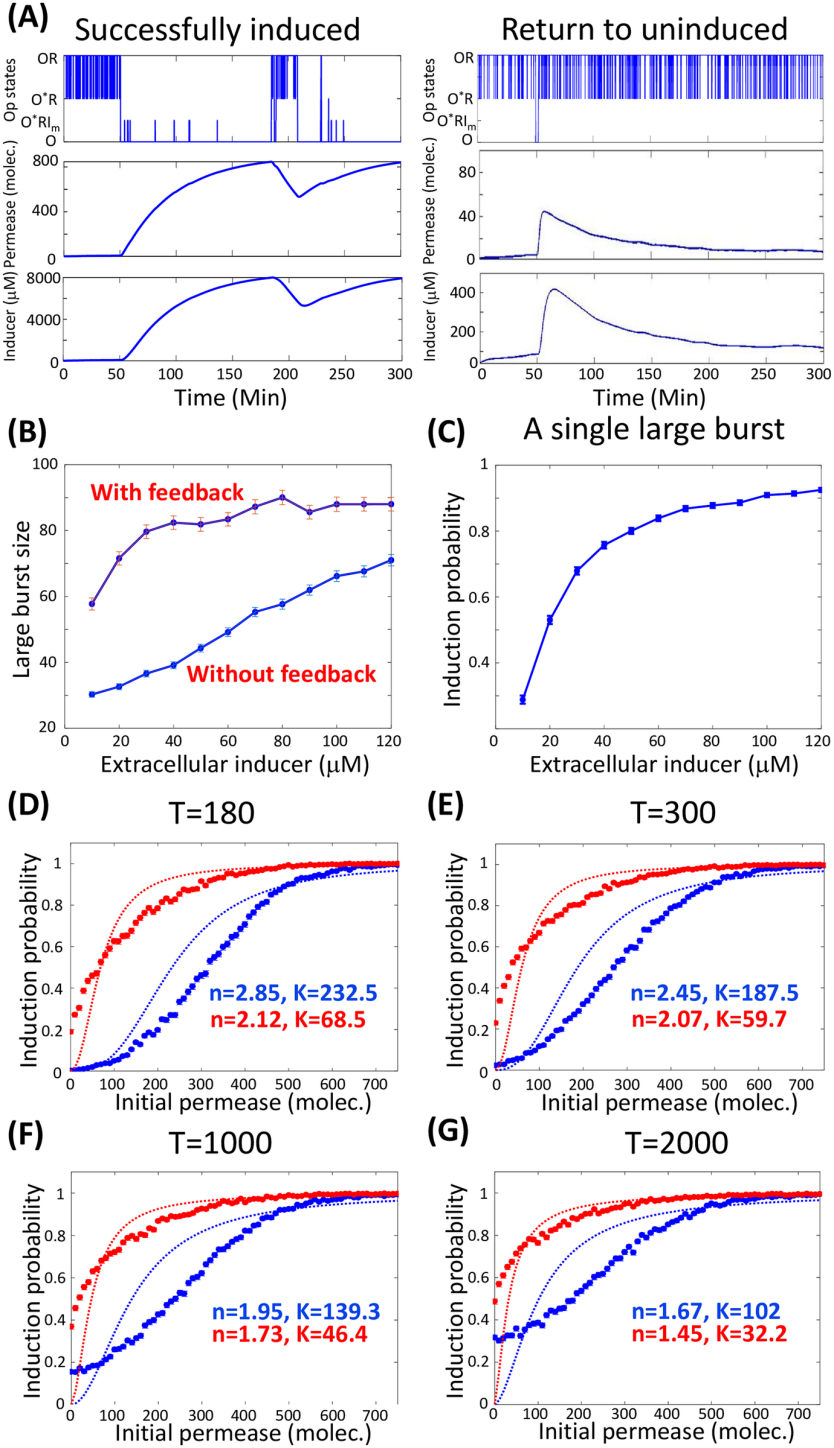
Probability of induction by a single large burst and quasi-steady state. (A)Two typical single-cell time traces of permease levels. The first shows induction by a single full dissociation event of the repressor from the operon (left), while the second shows a failure to induce (right). (B) The large burst size in the presence of positive feedback is remarkably prolonged compared with the case without positive feedback. (C) Successful probability of induction by a complete dissociation event as a function of the extracellular inducer concentration. (D-G) Probability of induction within different time windows starting from uninduced cells (blue) or induced cells (red); we determined the stochastic threshold through mathematical fitting in the form of $y=\frac{x^{n}}{x^{n}+K^{n}}$ for these curves. The deterministic threshold is approximately 20(*molec.*), while the stochastic thresholds are larger and decrease when the time window is extended. The extracellular inducer concentration, *I*_*e*_, is set to 40*μ*M.

How does a stochastic single-molecule event (i.e., a large burst) trigger phenotype transition? Time traces of permease showed that the stochastic full dissociation of the repressor from the DNA could either successfully trigger phenotype transition or return to the uninduced state before arriving at the induced state (Fig 5A). The positive feedback mechanism of the wild-type cell can significantly amlify the large burst, which dramatically increases the probability of transition from the uninduced to the induced state (Fig 5B and S7 Fig). We calculated the probability of successful induction of single cells initially in the uninduced state after a single-molecule event(i.e., the repressor completely dissociating from the operon), as a function of the extracellular inducer concentration (Fig 5C). Once the extracellular inducer concentration reached 40*μ*M, we found that the probability of induction was nearly 80%.

We were also interested in the transition from the induced to the uninduced state. Once the cell is induced, the intercellular concentration of the inducer is quite high, and the repressor would always choose another pathway to dissociate rapidly from the operon (*OR* → O*R → O*RI_*m*_ → *O* in Fig 1C). These events make the induced state much more stable than the uninduced state, and contribute to much smaller fluctuations around the induced state than around the uninduced state (Fig 4C and 4D). These predictions were also confirmed by our simulation (S8 Fig).

#### Stochastic threshold, time scales and quasi steady states

Another important quantity in a bistable system is the barrier or threshold between the two phenotypic states. The saddle point of the mean-field model is generally regarded as the “deterministic” threshold, which is an analog of the transition state in physical chemistry. In the presence of stochasticity, the definition of the threshold becomes vague.

Choi et al. measured the single-cell time traces of fluorescence, normalized by cell size, starting from different initial permease numbers, and they plotted the probability of induction within 3 hours as a function of the initial permease number [3]. Then the threshold is determined through a Hill-type function fitting.

However, the observed probability of induction in experiments is related to a quasi-steady state rather than the final steady state, because it depends on the initial permease number which is not a parameter but a dynamic variable of the system (Fig 2B in [3]). Because the transition rates between the different phenotypic states are low, the measured distribution is highly dependent on the time window of the experiments and the initial state. Using our model simulation, we rebuilt the experimental observations. We plotted the fraction of induced cells with different time windows starting from uninduced or induced cells (red and blue curves in Fig 5D–5G). The estimated threshold decreases with the extension of the experimental time window, and is different from the deterministic threshold predicted from the corresponding deterministic mean-field dynamics. It is clear that these two curves approach the same horizontal line over time, indicating that the final steady-state distribution is independent of both the time window and the initial state of the cell population.

The difference between the quasi-steady state and the final steady state reveals why the induced state dominates the final stationary distribution when the extracellular inducer concentration exceeds 40*μ*M, according to estimated transition rates (data not shown), but it is still possible to observe a distinct bimodal distribution at a reasonable time scale starting from uninduced states when the extracellular inducer concentration exceeds 40*μ*M (S2 Fig).

The difference between the quasi-steady state and final steady state increases in the region of deterministic bistability as the rate of gene-state switching increases. For example, when the gene-state switching rate is 100 times faster, within a certain time scale (time = 2000 min), cells will not become induced if they start in the uninduced state. However, it does exist on the other side of the deterministic threshold, whose stability could be clearly demonstrated if the cells start in the induced state (S9 Fig).

#### Transition rates between phenotypic states and resonance phenomena

Once the gene-state switching rates are not only slow compared to typical rates of active transcription and translation, but also rapid compared with the time scale of cell division, a general rate formula for phonotype transition in a fluctuating-rate model has been recently proposed [22]. The rate formula is associated with the phenotypic landscape function, which is an analog of the energy function at an equilibrium [22, 27, 39, 45–52].

The phenotypic landscape is defined as the negative logarithm of the steady-state probability distribution *p*^*ss*^(*x*) at the limit of infinite *ω* [22, 27, 39, 45, 47–50, 52], i.e.,

$$\begin{array}{c} \phi \left(x\right)=\lim_{\omega \to \infty }-\frac{1}{\omega }\log p^{ss}\left(x\right). \end{array}$$ (1)

**Fig 6:**
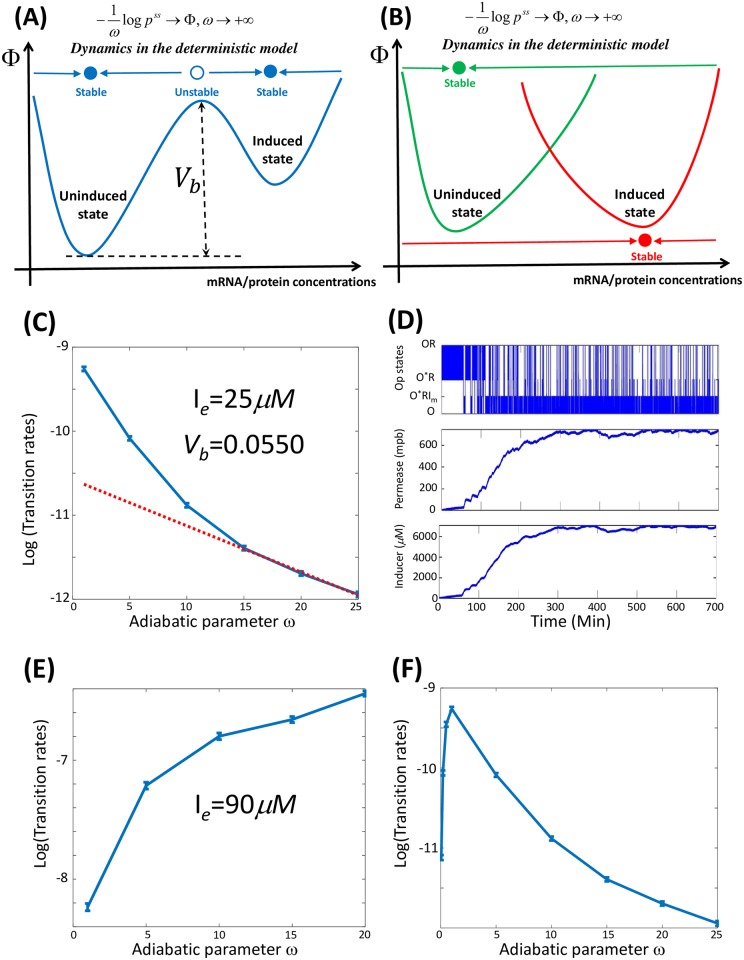
Transition rates between phenotypic states and the phenomenon of resonance. (A) Phenotypic landscape *ϕ*(*x*) in the region of deterministic bistability. (B) Phenotypic landscape *ϕ*(*x*) outside the region of deterministic bistability. (C) The rate formula (2) is valid for the parameter region of deterministic bistability with the fitted positive barrier *V*_12_ = 0.0550. (D) When the switching rates among different gene states are sufficiently rapid, the phenotype transition from the uninduced state to the induced state must occur through the accumulation of many complete dissociation events, rather than through a single dissociation event in wild-type cells, within the parameter region of deterministic bistability. (E) The transition rate increases and is finally saturated when the operon-state switching rate increases in the region of purely stochastic bistability. (F) The mean phenotype transition time varies with the operon-state switching rates at *I*_*e*_ = 25*μ*M.

Here, *ω* serves as a Boltzmann factor [*β* = (*k*_*B*_*T*)^−1^] in thermal physics. However, this deterministic landscape *ϕ*(*x*) is not given a priori; it is an emergent property of the chemical kinetics of a single cell. Furthermore, the most important feature of the function *ϕ*(*x*) is that the corresponding mean-field deterministic dynamics in the large limit of *ω*, always decrease along *ϕ*(*x*) (Fig 6A and 6B), which suggests that any local minimum of the function *ϕ*(*x*) corresponds to a stable steady state of the deterministic model [22, 47]. Therefore, the necessary and sufficient condition for deterministic bistability(i.e., two stable steady states predicted by the mean-field model) is a double-welled deterministic landscape *ϕ*(*x*) (Fig 6A and 6B).

Although the landscape function is not easily obtained, the most important consequence is the transition rate formula from the *i*-th phenotype to the *j*-th one [22], i.e.,

$$\begin{array}{c} k_{ij}≃k_{ij}^{0}e^{-\omega V_{ij}}, \end{array}$$ (2)

where the positive quantity *V*_*ij*_ is referred to as the barrier term from the i-th phenotypic state to the j-th phenotypic state, and $k_{ij}^{0}$ is a prefactor with units, all of which are independent of *ω*. From a dynamic perspective, this formula could also be understood through Kramers’ rate theory, in which *ω* ↔ (*k*_*B*_*T*)^−1^ is proportional to the reciprocal of the fluctuation amplitude, and a small *T* and large *ω* both represent small fluctuations. Theoretically, in the case of bistability, the barrier terms *V*_12_ and *V*_21_ in formula (2) are the minimum of the differences in the local maximum and minimum values, respectively, of the deterministic landscape *ϕ* along any transition path between the two phenotypic states [22].

Formula (2) is valid within the parameter region of deterministic bistability when *ω* is large. In this region, both phenotypic states are preserved within the large limit of *ω*. Additionally, the forward and backward barriers between the phenotypic states are positive, and the transition rates therefore decrease exponentially with *ω* (Fig 6C). The transition rate from the induced state to the uninduced state (∼ 10^−7^ min^−1^) is much lower than the forward transition rate (∼ 10^−4^ min^−1^), which is beyond our computational capacity. However, due to established mathematical theory [22, 53], the rate formula (2) is still valid.

We found that the simulated stochastic transit time from the uninduced state to the induced state exhibited an exponential distribution (S11 Fig). Additionally, when the gene-state switching rates were sufficiently rapid, the typical transition pathway from the uninduced state to the induced one is not the same as that of the wild-type by a single-molecule event, but due to the accumulation of many times of full dissociation events (Fig 6D).

Alternatively, in the parameter region of stochastic bistability, the two phenotypic states merge within the large limit of *ω*, and the bimodal distribution gradually becomes unimodal (S10 Fig). Thus, the transition between the two phenotypic states becomes relaxing towards one unique phenotypic state. Accordingly, the transition rate increases with *ω* and reaches a saturation value (Fig 6E), which is qualitatively different from the region of deterministic bistability.

We then numerically calculated the mean transition time dependent on the parameter *ω* in the parameter region of deterministic bistability when *I*_*e*_ = 25*μ*M. We showed that the transition rate from the uninduced state to the induced state reaches a maximum when the gene-state switching rates are around the wild-type values (Fig 6F), which is referred to as a resonant phenomenon [27, 42]. This occurs because, according to the rate in formula (2), the transition rate between phenotypic states decreases exponentially when gene-state switching is rather rapid. Meanwhile, when gene-state switching is very slow, it becomes the rate-limiting step for the phenotype transition, which is also extremely low.

## Discussion

Only a single copy of a DNA molecule exists inside a typical cell. Hence, the stochastic dynamics of a single cell resulting from the fluctuating kinetics of single DNA molecule are a consequence of fundamental physical and chemical laws. Still, individual cells can control the stochastic kinetics of DNA molecules over a reasonable time scale and fluctuations due to biochemical reactions can be even advantageous. Recent high-precision measurements performed in single cells have revealed that stochastic gene-state switching is slow compared to typical rates of active transcription and translation. The fluctuating-rate model is a good candidate for the investigation of single-cell dynamics in this region because it only incorporates the gene-state switching mechanism. The present study reveals the power of this type of model, which will be used to investigate other questions regarding single-cell dynamics.

We investigated the origin of the bimodal distribution of the *lac* operon in a realistic model incorporating the recently discovered mechanism of operon-state switching. It has been shown that either positive feedback or single-molecule fluctuations gives rise to bistability by its own. However, we show here that the interplay of these two mechanisms makes the bimodal distribution more realistic and reliable in the presence of environmental perturbations. Without positive feedback, the single-molecule kinetics of gene states are not sufficient slow, at least in *E. coli*, to induce bistability, and without fluctuations of single DNA molecules, positive feedback cannot stabilize the uninduced state when the extracellular inducer concentration is high. The physiological region for the gene-state switching rates is therefore favorable and balances the two contradictory purposes of controlling stochasticity within a certain magnitude and triggering phenotype transitions within a reasonable time scale.

The stochastic model can quantify the relative stability (fractions in a population) of coexisting phenotypes, which cannot be achieved using a deterministic approach. However, the time scale of the stochastically triggered spontaneous phenotype transition is quite long, which prevents direct laboratory measurement of the relative stability (given the time window of a typical experiment), due to inconsistencies between a quasi-steady state and the final steady state. Such inconsistencies also mean that the concept of the threshold is not well-defined in a stochastic scenario, which is considerably different from the deterministic threshold predicted from the deterministic mean-field model.

Recently, Razooky, et al. also investigated the interplay between positive feedback and relatively slow gene-state switching kinetics in the transcriptional program controlling HIV’s fate decision between active replication and viral latency, and found out that the positive feedback shifts and expands the region of LTR bimodality [54]. However, the positive feedback in LTR dynamics lacks cooperativity and cannot produce deterministic bistability by itself, which is essentially different from the *lac* operon dynamics we studied here. Also the perspective we used to explain the broadened bistability is different from [54]: their explanation more focused on the mean-noise relation while ours more focus on the stability of each phenotypic states.

In many experiments, people used minimal media for cells at 37°*C*, making the *E. coli* cells grow slowly (doubling time is about one hour). In there experiments, a single copy or at most two copies are reasonable. However, in a more natural environment, *E. coli* grows at most commonly 20 − 30 minutes doubling time, which implies more copies of operons. Hence we also simulate the case with more than one copy of operons (see Materials and methods), and find out that the qualitative results are exactly the same as the case with only a single copy of operon (S13 Fig).

On the other hand, in the main text, we model the *lac* operon under unnatural conditions, i.e. using the unnatural lactose-analogue TMG, which is used in most of the experiments. We also simulate the extended version of the model with lactose replacing TMG, in which an additional term representing the hydrolysis of allolactose is added (see Materials and methods). Under steady-state condition of extracellular lactose, the results are quite the same as those from the main model in which we use TMG (S15 Fig).

Finally, the notion of cell diversification of genetically identical phenotypes in biological entities, due to stochastic gene expression, requires a mechanism for the inheritance of an “intercellular biochemical” state through cell division. This issue has been discussed previously [55, 56]. Briefly, if the volume of a biochemical system doubles while maintaining the same internal concentrations, the phenotypic state of the cell is maintained. Therefore, the phenotype of a single cell can be preserved via growth and division into two daughter cells. This epigenetic inheritance mechanism is based on dynamic biochemical self-organization, which is fundamentally different from the Watson-Crick genetic template-copying mechanism.

## Materials and methods

### Model development

#### Mean-field deterministic model

The model consists of several differential equations that account for the temporal evolution of mRNA (*M*), the LacY polypeptide (*Y*), and the intracellular inducer concentration.

Here we take *R* to denote the concentration of the active (free) repressor while *R*_*T*_ denotes the total concentration of the repressor. Additionally *I*_*e*_ denotes the extracellular concentration of thiomethyl *β*-D-galactoside (TMG), and *I* denotes the intracellular TMG concentration.

The kinetic equations for the *lac* operon are

$$\begin{array}{ccc} \frac{R}{R_{T}} & = & \frac{K}{K+I^{n}},\\ \frac{dM}{dt} & = & k_{M}p_{O}-\gamma_{M}M,\\ \frac{dY}{dt} & = & k_{Y}M-\gamma_{Y}Y,\\ \frac{dI}{dt} & = & \alpha k_{l}Y\beta \left(I_{e}\right)-\gamma_{I}I+c\left(I_{e}-I\right). \end{array}$$ (3)

where *p*_*O*_ is the probability that the operon is free. Traditionally, *p*_*O*_ is expressed as $\frac{a}{1+\frac{R}{R_{0}}}=a\frac{K+I^{n}}{K+I^{n}+\frac{R_{T}}{R_{0}}K}$, where *a* is the highest probability that can be archived when *R* = 0, and *R*_0_ is the half saturation concentration of the repressor bound to TMG; however, after taking the partial dissociation state into account in the present study, we believe that it should be modified (See Eq (6) and S1 Text). The Hill coefficient *n* is approximately 2 according to experimental measurements [4]. Although there are 4 subunits in the repressor protein, *K* is the equilibrium constant of the binding reaction between the repressor and inducer when not bound to the operon. *k*_*M*_ is the maximum transcription rate and *γ*_*M*_ represents the degradation plus dilution rate. *k*_*Y*_ is the initial rate of translation for LacY transcripts. *γ*_*Y*_ is the dilution and degradation rate of LacY polypeptides.

The variable *α*, which has two values, 0 or 1, denotes whether LacY is replaced by Tsr and positive feedback is absent. The inducer could diffuse into the cell quickly, even in the absence of permease; therefore, we denote *c* as the diffusion constant due to the difference in the inducer concentrations across the cell membrane. The inducer (TMG) could be also transported into the bacterium via a catalytic process in which permease plays a central role. Thus, the inducer influx rate is assumed to be *k*_*I*_*β*(*I*_*e*_)*Y*. *γ*_*I*_ is the dilution and degradation rate of the inducer. Here the form of *β*(*I*_*e*_) is taken from [4],

$$\begin{array}{ccc} \beta (I_{e}) & = & I_{e}^{0.6}. \end{array}$$ (4)

The first equation in Eq 3 indicates that the kinetics of the repressor binding to TMG are rapid; thus its kinetics yield an instantaneous fraction of the free repressor to total repressor (rapid-equilibrium assumption) [4, 30].

As the concentration of TMG varies, the system generates either one or two stable steady states, with a saddle-node bifurcation that separates the two phases. The existence of two stable steady states is in accord with the all-or-none phenomenon observed in both population and single-cell experiments [3, 57], by which we mean that a cell can exist in only one of the two phenotypic states. The population behavior varies due to changes in the relative portion of the two states, but no cell can exist in an intermediate state.

#### Single-molecule fluctuating-rate model

The mean-field approach neither explains the most recent experimental observations nor is consistent with the proposed stochastic mechanism [3]. Therefore we developed a stochastic model that incorporates the stochastic single-molecule operon-state switching.

Most cells possess only one or two copies of any given gene. Hence, we separated the time scales for operon-state switching and other evolutionary processes in the system, to introduce a stochastic variable, *η*, which accounts for the regulation of transcriptional initiation by active repressors. Single-molecule events(i.e., whether the operon is bound or unbound to a repressor(s)) can be modeled by a simple Markovian jumping process (i.e., rate equations; see Fig 1B and 1C).

The switching rates between different operon states in wild-type cells are estimated and provided in the S1 Text. We multiply each gene-state switching rate in Fig 1B and 1C by a non-dimensionalized number, *ω*, which describes how rapid the switching rates are compared with the wild-type. The parameter *ω* plays a central role in the investigation of phenotype-transition in the main text.

In Fig 1B and 1C, O*R and O*RI_*m*_ denote the partial dissociation state of DNA. Hence the variable *η* is a stochastic trajectory that has only three values, 0, *f* and 1, which denote the transcriptional levels when fully repressed (*OR*), partially dissociated (O*R and O*RI_*m*_) and completely dissociated *O*, respectively.

Finally, we obtain a three-dimensional differential equation that contains the variable *η* mentioned above.

$$\begin{array}{ccc} \frac{dM}{dt} & = & k_{M}\eta -\gamma_{M}M,\\ \frac{dY}{dt} & = & k_{Y}M-\gamma_{Y}Y,\\ \frac{dI}{dt} & = & \alpha k_{l}Y\beta \left(I_{e}\right)-\gamma_{I}I+c\left(I_{e}-I\right). \end{array}$$ (5)

In addition, the term *p*_*O*_ in the deterministic model (3) is the mean of *η*:

$$\begin{array}{c} p_{O}=\left\langle \eta \right\rangle =\frac{1+f(K_{1}\left[R\right]+K_{1}K_{3}\left[R\right]I^{n})}{1+K_{1}\left[R\right]+K_{1}K_{3}\left[R\right]I^{n}+K_{1}K_{2}\left[R\right]}=\frac{K+I^{n}+f\left(KK_{1}R_{T}+\frac{R_{T}}{K_{4}}I^{n}\right)}{K+I^{n}+K\left(K_{1}+K_{1}K_{2}\right)R_{T}+\frac{R_{T}}{K_{4}}I^{n}}, \end{array}$$ (6)

where *K*_*i*_ = *r*_*i*_/*r*_−*i*_. Each repressor head is a dimer and can bind 0, 1, or 2 inducer molecules, and we set *n* ≃ 2 due to cooperativity [4].

### Extended versions of the model

Several extended versions of the fluctuating-rate model have also been investigated: (1) Without feedback: set *α* to be zero; (2) Without DNA loop: there is only two operon states *O* and *OR*; (3) In the case of multiple operons: independent *n* operons coupled only through the intracellular *M*, *Y*, *I*, and the corresponding cell division time is set to be 50/*n* minutes, which makes the parameters *r*_*I*_ = 0.012*n*min^−1^ and *r*_*Y*_ is equal to 0.1 + *r*_*I*_; (4) Lactose replacing TMG: a term $-hyd\cdot \frac{I}{I+K_{I}}\cdot Y$ representing the hydrolysis of allolactose is added to the right-hand-side of $\frac{dI}{dt}$. Simulated results from these extended versions are in the Supporting Information.

### Quantifying bistability in the presence of stochasticity

We define the bimodal steady-state distribution as bistability in the presence of stochasticity. However, it is quite time-consuming to obtain the exact steady-state distribution in simulation, if it is bimodal. Luckily, if we only want to determine whether the steady-state distribution is bimodal or not, it is much easier. There is a fact that if the steady-state distribution is unimodal, the simulated distribution will rapidly converge to the steady-state distribution, while if the steady-state distribution is bimodal, the converging time is extremely long. Hence, we can use the quasi-steady-state distribution and hysteresis response curves to determine whether the system is bistable or not. We only need to simulate the system for a reasonably long time, which is enough for making the system converge into the unimodal steady-state distribution if it is not bistable, or into the bimodal quasi-steady-state distribution if it is bistable. Hysteresis response curve follows the same idea. After a reasonably long time, if the simulated distributions starting from induced state or uninduced state can not merge together, then it implies bistability.

### Stochastic simulation method

We used the standard exact method to simulate the dynamics of the operon developed by Doob, Bortz et al., and Gillespie [58–61]. See the Supplementary Material in [22] for details.

## Supporting information

S1 Text Mathematical derivations and calculations of parameters. (PDF)

S1 Fig A two-state model of the central dogma without feedback. (TIF)

S2 Fig Copy-number distributions for the permease protein in wild-type cells. We compare the copy-number distribution of permease with different extracellular concentration of inducers *I*_*e*_ and show that the *I*_*e*_ range of the bimodal distribution is much more broader than that predicted in the deterministic bifurcation diagram(Fig 2A in the main text).
(TIF)

S3 Fig Broad copy-number distributions for permease protein without positive feedback. (TIF)

S4 Fig Copy-number distributions for permease protein under different values of *I*_*e*_ when *k*_*M*_ is small. (TIF)

S5 Fig Copy-number distributions for permease protein under different values of *I*_*e*_ when *k*_*M*_ is large. (TIF)

S6 Fig Size and frequency of small and large bursts without positive feedback, dependent on the extracellular inducer concentration. (TIF)

S7 Fig Copy-number distribution for the newly synthesized permease protein during a single large burst with positive feedback, which is quite similar to exponential distribution. (TIF)

S8 Fig Will a single repressor rebinding event trigger the phenotype transition from the induced state to the uninduced state? The uninduction probability nearly vanishes when the extracellular inducer concentration is only slightly larger than about 40*μM*.
(TIF)

S9 Fig Copy-number distributions for the permease protein observed in the region of deterministic bistability, varying with *ω*. (TIF)

S10 Fig Copy-number distributions for the permease protein observed in the region of purely stochastic bistability, varying with *ω*. (TIF)

S11 Fig Nearly exponentially distributed transition time from the uninduced state to the induced state in wild-type cells. (TIF)

S12 Fig Stochastic hysteresis response of the probability of induction when tuning the strengths of stochasticity. Initial conditions: uninduced (blue line) or fully induced (red line) cells with a period of *T* = 2000 min.
(TIF)

S13 Fig Bistability with and without stochastic operon-state switching when the number of operons are more than one. (A)(B) Deterministic bifurcation diagram for wild-type cells in which the number of operons is 2 or 6. (C)(D) Deterministic bifurcation diagrams for the repressor bound to the operon in the absence of a DNA loop with association constant that equals 5 *molec.*^−1^. (E) (F) Stochastic hysteresis response of the probability of induction.
(TIF)

S14 Fig Bistability with and without stochastic operon-state switching tuning the strength of positive feedback. (A)(B) Deterministic bifurcation diagram tuning the strength of positive feedback. (C-F) Stationary distributions when tuning the strength of positive feedback.
(TIF)

S15 Fig Bistability with and without stochastic operon-state switching when the dynamics of inducer is replaced by that of lactose. (A-D) Deterministic bifurcation diagram in which the dynamics of inducer is replaced by that of lactose. (E) (F) Stochastic hysteresis response of the probability of induction.
(TIF)

S1 Table Values of kinetic parameters in the fluctuating-rate model. (PDF)
