# Supplementary material for: Relatively slow stochastic gene-state switching in the presence of positive feedback significantly broadens the region of bimodality through stabilizing the uninduced phenotypic state
Source: PLoS Comput Biol. 2018 Mar 12;14(3):e1006051. doi: 10.1371/journal.pcbi.1006051 (PMC5864076; doi:10.1371/journal.pcbi.1006051)
Supplement: S1 Text — (PDF) [file pcbi.1006051.s001.pdf]

# Supporting Information: Relatively slow stochastic gene-state switching in the presence of positive feedback significantly broadens the region of bimodality through stabilizing the uninduced phenotypic state

Hao Ge[1,2*] Pingping Wu[3] Hong Qian[4] and X. Sunney Xie[1,5*]

[1]Biodynamic Optical Imaging Center(BIOPIC)
Peking University, Beijing 100871, P.R.China
and
[2]Beijing International Center for Mathematical Research (BICMR)
Peking University, Beijing 100871, P.R.China
and
[3]School of Mathematical Sciences
and Centre for Computational Systems Biology
Fudan University, Shanghai 200433, P.R.China
and
[4]Department of Applied Mathematics
University of Washington, Seattle, WA 98195, USA
and
[5]Department of Chemistry and Chemical Biology
Harvard University, Cambridge, MA 02138, USA

March 6, 2018

* Corresponding authors
E-mail: haoge@pku.edu.cn(HG); xie@chemistry.harvard.edu(XSX)

# 1 Stochastic models of central dogma

## 1.1 Simplest mechanism: a single DNA state

A single DNA waits for an exponential distributed time window with parameter $k_1$ until it gives rise to a piece of mRNA, and then the life time of each $mRNA$ also obeys an exponential distribution with parameter $d_1$. Within the life time of each $mRNA$, protein $P$ is being synthesized by a Poisson process with intensity $k_2$, and at the same time, each molecule of $P$ degrades with parameter $d_2$.

During each single burst, the distribution of synthesized protein is geometric with parameter $q = \frac{k_2}{k_2+d_1}$. More precise,

$$G_n = \int_0^\infty d_1 e^{-d_1 t} e^{-k_2 t} \frac{(k_2 t)^n}{n!} dt = q^n (1-q).$$

Its average is $q/(1-q)$, which is regarded as the burst size. This fact was first proved by [12] and recently confirmed by experiments [14].

And the master equation for the population distribution of protein is

$$\frac{dP(n,t)}{dt} = k_1 (\sum_{j=1}^{n} G_j P(n-j,t) - qP(n,t)) + d_2((n+1)P(n+1,t) - nP(n,t)),$$

whose stationary distribution is just the negative binomial [16]

$$P^{ss}(n) = \frac{b^n}{(1+b)^{a+n}} \frac{\Gamma(a+n)}{\Gamma(a)n!},$$

where $a = \frac{k_1}{d_2}$ is the burst frequency per cell cycle and $b = \frac{k_2}{d_1} = q/(1-q)$ is just the burst size. $a$ and $b$ are typically determined through the fitting of the stationary distribution that measured.

Interestingly, it is not the real burst size observed if you track the protein in a single cell, because one could not observe $G_0$ (although $P^{ss}(n)$ could be observed), hence this kind of real burst size $\tilde{b} = b/(1-G_0) = b+1$.

## 1.2 Two-state model

See Fig S1. Assume the parameters for the exponential distributed "on" time of the DNA and mRNA are $\beta$, $d_1$ respectively, and within that "on" time, the mRNA and protein are being synthesized, by another two Poisson process with intensities $k_1$ and $k_2$ repectively.

It is easy to prove that the number of mRNA synthesized per single "on" period of DNA and the number of newly synthesized protein per single "on" period of mRNA both obey geometric distribution with parameter $\theta_1 = \frac{k_1}{\beta+k_1}$ and $\theta_2 = \frac{k_2}{d_1+k_2}$ respectively.

Therefore, the distribution of newly synthesized protein number during a single "on" period of DNA is

$$G_n = \sum_{k=1}^{\infty} \theta_1^k (1-\theta_1) \sum_{n_1+n_2+\cdots+n_k=n} \Pi_{i=1}^k (\theta_2)^{n_i}(1-\theta_2), \ n \geq 1,$$

and $G_0 = 1 - \theta_1 + \sum_{k=1}^{\infty} \theta_1^k (1-\theta_1)(1-\theta_2)^k$.

Since the function $f(n,x) = \sum_{k=1}^{\infty} x^k \frac{(n+k-1)!}{n!(k-1)!}$ satisfies $f(n,x) = \frac{f(n-1,x)}{1-p}$, then $f(n,x) = \frac{x}{(1-x)^{n+1}}$.

Therefore,

$$G_n = \sum_{k=1}^{\infty} \theta_1^k (1-\theta_1) \frac{(n+k-1)!}{n!(k-1)!} (\theta_2)^n (1-\theta_2)^k = \theta_1 \frac{(1-\theta_1)(1-\theta_2)}{1-\theta_1+\theta_1\theta_2} (\frac{\theta_2}{1-\theta_1+\theta_1\theta_2})^n, \ n \geq 1,$$

and $G_0 = 1 - \frac{\theta_1\theta_2}{1-\theta_1+\theta_1\theta_2}$.

This is a modified geometric distribution with parameter $\theta_1$ and $\theta_2$. In this case, we don't think experimentally one can tell the difference between this and a real geometric distribution since all the difference is in the distributions at $n = 0$ and $n = 1$, overwhelmed in the experimental noise.

Consequently, the averaged value of this modified geometric distribution is

$$\langle n \rangle = \sum_{n=1}^{\infty} n G_n = \frac{\theta_1\theta_2}{(1-\theta_1)(1-\theta_2)} = \frac{k_1 k_2}{d_1 \beta}.$$

The master equation for the population distribution of protein under the bursty condition is

$$\frac{dP(n,t)}{dt} = \alpha \frac{\beta}{\alpha+\beta} (\sum_{j=1}^{n} G_j P(n-j,t) - \sum_{j=1}^{\infty} G_j P(n,t)) + d_2((n+1)P(n+1,t) - nP(n,t)),$$

where $G_j = \theta_1 \frac{(1-\theta_1)(1-\theta_2)}{1-\theta_1+\theta_1\theta_2} (\frac{\theta_2}{1-\theta_1+\theta_1\theta_2})^j$.

Its stationary distribution is just the negative binomial

$$P^{ss}(n) = \frac{b^n}{(1+b)^{a+n}} \frac{\Gamma(a+n)}{\Gamma(a)n!},$$

where $a = \frac{k_1}{d_2}\frac{\beta}{\alpha+\beta}\frac{\alpha}{\beta+k_1}$ is the burst frequency and $b = \frac{\theta_2}{(1-\theta_1)(1-\theta_2)} = \frac{k_2}{d_1}(\frac{k_1}{\beta}+1)$ is the burst size. Notice that $b$ could not be less than $\frac{k_2}{d_1}$ which is the mean translated protein number per mRNA molecule.

The real burst size if you track the protein in a single cell $\tilde{b} = \langle n \rangle/(1-P(0)) = \frac{k_1 k_2}{\beta d_1} + \frac{k_2}{d_1} + 1 = b + 1$.

If $\beta \gg \alpha, k_1$, then the two-state model can be approximated by the simplest model with only one gene state, since the rate limiting step to synthesize a new mRNA is the switching from the inactive state to the active state. In this case, the equally $\tilde{k_1} = \frac{k_1\alpha}{\alpha+\beta}$. Then the burst frequency and size in the two-state model reduce to those in the simplest model.

## 1.3 Applied to modified repressor-dependent model with DNA loop

**Small burst**

During each single small burst, the model could be reduced to

$$O^*R \underset{r_{-2}}{\overset{r_2}{\rightleftharpoons}} OR,$$

in which $OR$ and $O^*R$ are the gene states with the repressor bound to both operators or only the weaker one. Here $k_1 = fk_M$, $k_2 = k_Y$, $d_1 = \gamma_M$ and $d_2 = \gamma_Y$.

Therefore, the population distribution of protein is the negative binomial with parameters $a_{small} = \frac{fk_M}{\gamma_Y}\frac{r_2}{r_2+r_{-2}}\frac{r_{-2}}{r_2+fk_M}$ and $b_{small} = \frac{fk_M k_Y}{r_2\gamma_M} + \frac{k_Y}{\gamma_M}$.

**Large burst in the absence of positive feedback**

When the inducer concentration is low, then Fig 1C in the maintext could be approximated by

$$O + R \underset{r_{-1}}{\overset{r_1}{\rightleftharpoons}} O^*R \underset{r_{-2}}{\overset{r_2}{\rightleftharpoons}} OR,$$

where $O^*R$ denotes the partial dissociation state of DNA.

Here $k_1 = k_M$, $k_2 = k_Y$, $d_1 = \gamma_M$ and $d_2 = \gamma_Y$.

The burst size is easy to compute as $b_{large} = \frac{k_M k_Y}{\gamma_M r_1[R]} + \frac{k_Y}{\gamma_M}$, and the frequency could be computed by the first passage time starting from the state $OR$ to $O$, i.e. $\langle T \rangle = \frac{r_{-1}+r_2+r_{-2}}{r_{-1}r_{-2}} \approx \frac{K_2+1}{r_{-1}}$ since $r_2, r_{-2} \gg r_{-1}$, hence $a_{large} = \frac{k_M}{\gamma_Y}\frac{r_1[R]}{r_1[R]+\frac{1}{\langle T \rangle}}\frac{\frac{1}{\langle T \rangle}}{r_1[R]+k_M} \approx \frac{k_M}{\gamma_Y}\frac{\frac{r_{-1}}{K_2+1}}{r_1[R]+k_M}$.

Hence both the large burst frequency and size increase with intracellular inducer concentration, but the frequency will not change that much while the size would increase significantly.

When the inducer concentration is high, then Fig 1C in the main text could be approximated by an alternative pathway

$$O + RI_m \underset{r_4}{\overset{r_{-4}}{\rightleftharpoons}} O^*RI_m \underset{r_3 I^n}{\overset{r_{-3}}{\rightleftharpoons}} O * R \underset{r_{-2}}{\overset{r_2}{\rightleftharpoons}} OR.$$

The burst size $b_{large} = \frac{k_M k_Y}{\gamma_M r_{-4}[RI_m]} + \frac{k_Y}{\gamma_M} \approx \frac{k_M k_Y}{\gamma_M r_{-4} R_T} + \frac{k_Y}{\gamma_M}$. The burst frequency is approximately $a_{large} = \frac{k_M}{\gamma_Y} \frac{r_{-4}[RI_m]}{r_{-4}[RI_m]+r_3 I^n/(K_2+1)} \frac{r_3 I^n/(K_2+1)}{r_{-4}[RI_m]+k_M}$.

Hence the large burst size is nearly constant, and the frequency will slightly increase with intracellular inducer concentration with the limit $\frac{k_M}{\gamma_Y} \frac{r_{-4} R_T}{r_{-4} R_T + k_M}$.

However, as we have mentioned in the main text, the bursts would be much more indistinct when the intracellular inducer concentration is quite high.

For intermediate intracellular inducer concentration, the general expression of large burst size can be well approximated by

$$b_{large} = \frac{k_M k_Y}{(r_1[R] + r_{-4}[RI_m])\gamma_M} + \frac{k_Y}{\gamma_M}.$$

## 2 Calculation of parameters

First of all, based on Fig 1 of the main text, we assume that the system satisfies the thermodynamic constrain:

$$r_1[R] r_3 I^n r_4 = r_{-1} r_{-4} [RI_n] r_{-3},$$

and $K = \frac{[R]I^n}{[RI_n]}$, hence

$$K = \frac{r_{-1} r_{-3} r_{-4}}{r_1 r_3 r_4} = \frac{1}{K_1 K_3 K_4}.$$

Then we could get that

$$p_O : p_{O*R} : p_{O^*RI_n} : p_{OR} = 1 : K_1[R] : K_1 K_3 [R] I^n : K_1 K_2 [R],$$

where $K_3 = \frac{r_3}{r_{-3}}$. Therefore,

$$p_O = \frac{1 + f(K_1[R] + K_1 K_3 [R] I^n)}{1 + K_1[R] + K_1 K_3 [R] I^n + K_1 K_2 [R]} = \frac{K + I^n + f(K K_1 R_T + \frac{R_T}{K_4} I^n)}{K + I^n + K(K_1 + K_1 K_2) R_T + \frac{R_T}{K_4} I^n}.$$

**Lac operon copy number and production/degradation rates**

Kennell measured the translation rate of LacZ as $18.8 min^{-1}$ [30] and (Kennell and Riezman, 1977), which is also consistent with [14]. And since LacZ is nearly expressed 10

times higher than LacY, hence we set transcription rate of LacY as $k_Y = 1.8min^{-1}$. The mRNA half-life time for LacA is 55 seconds according to (Kennell and Riezman, 1977), hence $\gamma_M = \ln 2/(55/60) = 0.756min^{-1}$. Also Tsr and permease should have similar rates. In fact, Choi, et al. [3] did a direct comparison of them (LacY-Venus replaced by Tsr-Venus) and find similar expression levels.

The cell-doubling time is about 60 minutes, hence the dilution rate for the stable protein TMG $\gamma_I = 0.012min^{-1}$. And the Lac permease degradation rate is about $0.01min^{-1}$, then $\gamma_Y = 0.01 + 0.012 = 0.022min^{-1}$.

The in-vivo transcription rate of LacY $k_M$ is more complicated. Kennell's estimate of $k_M$ in vitro is closer to $18min^{-1}$. However, for the fully induced cells, the probability of open operon is nearly 1 and the mRNA should approximately $k_M/\gamma_M \approx 23.8$, which is $2-3$ folds of that observed recently (almost every gene has an mRNA copy number of less than 10 per cell in [20]). This value also varies: for instance, $k_M \approx 9min^{-1}$ in (Chung and Stephanopoulos, 1996) determined from theoretical fitting, and even simple physical or chemical data published by different groups will differ by factors of $2-10$. So in order to become more quantitatively consistent with recent experiments, we here set $k_M = 8min^{-1}$.

**Parameters for operon stochastic dynamics**

According to Elf, et al.'s kinetic measurement in vivo (Elf et al., 2007), there are about $1-3$ repressers per cell, and each repressor will spend about $5-6$ minutes to rebind, hence $R_T = 2molec.$ and the association rate $r_1 \approx 0.2molec.^{-1}min^{-1}$. Referred to the in-vitro measurements of Table I and III of [31], the binding constants for repressor and inducer $\sqrt{K}$ is about $10\mu M$, and that for repressor/operon and inducer $\frac{1}{\sqrt{K_3}}$ is several mM (Matzke et al., 1992). However, in the in-vivo experiments, it seems that $K = 100molec.^{-1}\mu M^2$ is too low since the uninduced cells still should exist when the intracellular inducer concentration increases to nearly $50-100\mu M$. The inconsistence of the in vitro and in vivo measurement might result from the much more complicated environment inside a living cell. For example, the DNA-protein interactions are highly salt-dependent, and the flexibility and persistence length of DNA in vivo does not seem to match in vitro values, probably because there are other proteins binding to the DNA that can cause bends, etc. Therefore, we modify $K$ to be $2500molec.^{-1}\mu M^2$ and set $K_3 = 8 \times 10^{-6}\mu M^{-2}$. Then according to the thermodynamic constrain, we can have $K_1 K_4 = \frac{1}{KK_3} = 50molec.$.

Furthermore, the mRNA level/ protein level is nearly $k_Y/\gamma_Y \approx 100$, which is consistent with experimental observations for 1000 induction ratio (for uninduced cells, mRNA=0.01 and protein=1; for induced cells, mRNA=10 and protein=1000). It also implies the proba-

bility for unrepressed operon $p_O$ as the intracellular inducer concentration is low ($\leq 50\mu M$) is nearly 0.001, and the main pathway is

$$O + R \underset{r_{-1}}{\overset{r_1}{\rightleftharpoons}} O^*R \underset{r_{-2}}{\overset{r_2}{\rightleftharpoons}} OR,$$

hence $p_O \approx \frac{1+fK_1[R]}{1+K_1[R]+K_1K_2[R]} \approx \frac{f}{K_2+1} = 0.001$.

According to the classic measurements in (Oehler et al., 1990) and recent observations in (Garcia et al., 2012), the repression by only the distal operator is neglectable, hence we set $f = 1$ and $K_2 = 1000$. These approximation is reasonable since the small burst frequency without feedback $a_{small} = \frac{fk_M}{\gamma_Y}\frac{r_2}{r_2+r_{-2}}\frac{r_{-2}}{r_2+fk_M}$ in the case $r_2 \gg fk_M$ can be approximated by $a \approx \frac{fk_M}{\gamma_Y}/(K_2+1) = 0.363$, which is quite consistent with the observation in [3]. Further, the small burst size $b_{small} = \frac{fk_Mk_Y}{r_2\gamma_M} + \frac{k_Y}{\gamma_M} \approx \frac{k_Y}{\gamma_M} = 2.38$ as long as $r_2 \gg fk_M$, which is also consistent with the observation in [3].

We also require the large burst size at $100\mu M$ intracellular inducer concentration is about 100 molecules (Choi et al., 2010), i.e.

$$b_{large} = \frac{k_Mk_Y}{(r_1[R] + r_{-1}[RI_m])\gamma_M} + \frac{k_Y}{\gamma_M} \approx 100.$$

And the large burst frequency $a_{large} \approx 0.01$ when intracellular inducer concentration is $50\mu M$(Choi et al., 2010), i.e.

$$\begin{aligned} &\frac{k_M}{\gamma_Y}\frac{r_1[R]}{r_1[R] + \frac{r_{-1}}{K_2+1}}\frac{\frac{r_{-1}}{K_2+1}}{r_1[R] + k_M} \\ \approx &\frac{k_M}{\gamma_Y}\frac{\frac{r_{-1}}{K_2+1}}{r_1[R] + k_M} \approx 0.01. \end{aligned}$$

Taking into account all these requirements, we could set $K_1 = 5/8 molec.^{-1}$ and $K_2 = 1000$. Further $r_{-1} = 0.32\,\text{min}^{-1}$, $K_4 = 80 molec.$. $K_4$ is much higher than $1/K_1$, which is consistent with the fact that the repressor's affinity for the operator is substantially reduced to a level comparable to that of nonspecific DNA interaction. According to the observation that the inducer could begin to directly interact with repressed operon at about $200\mu M$, that is to say at this case, $r_{-1} = r_3(200)^n$. Hence $r_3 = 8 \times 10^{-6}\mu M^{-2}min^{-1}$, and $r_{-3} = 1\,\text{min}^{-1}$.

Finally, we require $r_2 = 1000r_{-2} \gg fk_M = 8$, and $r_4 = 80r_{-4} \gg 1$ in order to pull off repressor from the operator very rapidly when $I$ is sufficiently high. Hence we set $r_2 = 1000\,\text{min}^{-1}$, $r_{-2} = 1\,\text{min}^{-1}$ and $r_4 = 6\,\text{min}^{-1}$, $r_{-4} = 0.075 molec.^{-1}\,\text{min}^{-1}$. We know the induction time is $100min$ (about 0.6 cell cycle), and here when $I = 1000 - 1500\mu M$,

the burst frequency $a_{large} = \frac{k_M}{\gamma_Y} \frac{r_{-4}[RI_m]}{r_{-4}[RI_m]+r_3 I^n/(K_2+1)} \frac{r_3 I^n/(K_2+1)}{r_{-4}[RI_m]+k_M} \approx 0.35 \sim 0.7$, which is quite consistent.

**Inducer uptake rate**

Without feedback, the intracellular level will likely be equal to the extracellular, within 10 minutes at most , hence we could set $c \approx \ln 10/10 = 0.23 min^{-1}$.

And finally $k_l = 0.25 min^{-1}$ is determined to make the bistability range of our simulation roughly consistent with the experimental observation.

All parameters are summarized in S1 Table.

# References


[1] Choi PJ, Xie XS, Shakhnovich EI. Stochastic switching in gene networks can occur by a single-molecule event or many molecular steps. J. Mol. Biol. 2010; 396: 230-244.

[2] Chung JD, Stephanopoulos G. On physiological multiplicity and population heterogeneity of biological systems. Chem. Eng. Sci. 1996; 51:1509-1521.

[3] Elf J, Li GW, Xie XS. Probing transcription factor dynamics at the single-molecule level in a living cell. Science 2007; 316: 1191-1194.

[4] Garcia HG, Sanchez A, Boedicker JQ, Osborne M, Gelles J, Kondev J, et al. Operator sequence alters gene expression independentlu of transcription factor occupancy in bacteria. Cell Rep. 2012; 2(1): 150-161.

[5] Kennell D, Riezman H. Thanscription and translation initiation frequencies of the Escherichia coli Lac operon. J. Mol. Biol. 1977; 114: 1-21.

[6] Matzke EA, Stephenson LJ, Brooker RJ. Functional role of arginine 302 within the lactose permease of Escherichia coli. J. Biol. Chem. 1992; 267: 19095-19100.

[7] Oehler S, Eismann ER, Krämer H, Müller-Hill B. The three operators of the *lac* operon cooperate in repression. EMBO J 1990; 9(4): 973-979.


# 3 Supporting figures and table

**Fig S1** A two-state model of the central dogma without feedback.

**Fig S2** Copy-number distributions for the permease protein in wild-type cells. We compare the copy-number distribution of permease with different extracellular concentration of inducers $I_e$ and show that the $I_e$ range of the bimodal distribution is much more broader than that predicted in the deterministic bifurcation diagram(Fig. 2A in the main text).

**Fig S3** Broad copy-number distributions for permease protein without positive feedback.

**Fig S4** Copy-number distributions for permease protein under different values of $I_e$ when $k_M$ is small.

**Fig S5** Copy-number distributions for permease protein under different values of $I_e$ when $k_M$ is large.

**Fig S6** Size and frequency of small and large bursts without positive feedback, dependent on the intracellular inducer concentration.

**Fig S7** Copy-number distribution for the newly synthesized permease protein during a single large burst with positive feedback, which is quite similar to exponential distribution.

**Fig S8** Will a single repressor rebinding event trigger the phenotype transition from the induced state to the uninduced state? The uninduction probability nearly vanishes when the extracellular inducer concentration is only slightly larger than about $40\mu M$.

**Fig S9** Copy-number distributions for the permease protein observed in the region of deterministic bistability, varying with $\omega$.

**Fig S10** Copy-number distributions for the permease protein observed in the region of stochastic bistability, varying with $\omega$.

**Fig S11** Nearly exponentially distributed transition time from the uninduced state to the induced state in wild-type cells.

**Fig S12** Stochastic hysteresis response of the probability of induction when tuning the strengths of stochasticity. Initial conditions: uninduced (blue line) or fully induced (red line) cells with a period of $T = 2000$ min.

**Fig S13** Bistability with and without stochastic operon-state switching when the number of operons are more than one. (A)(B) Deterministic bifurcation diagram for wild-type cells in which the number of operons is 2 or 6. (C)(D) Deterministic bifurcation diagrams for the repressor bound to the operon in the absence of a DNA loop with association constant that equals 5 $molec.^{-1}$. (E) (F) Stochastic hysteresis response of the probability of induction.

**Fig S14** Bistability with and without stochastic operon-state switching tuning the strength of positive feedback. (A)(B) Deterministic bifurcation diagram tuning the strength of positive feedback. (C-F) Stationary distributions when tuning the strength of positive feedback.

**Fig S15** Bistability with and without stochastic operon-state switching when the dynamics of inducer is replaced by that of lactose. (A-D) Deterministic bifurcation diagram in which the dynamics of inducer is replaced by that of lactose. (E) (F) Stochastic hysteresis response of the probability of induction.

**Table S1** Values of kinetic parameters in the fluctuating-rate model.
