# Supplementary figures and images for: Relatively slow stochastic gene-state switching in the presence of positive feedback significantly broadens the region of bimodality through stabilizing the uninduced phenotypic state
Source: PLoS Comput Biol. 2018 Mar 12;14(3):e1006051. doi: 10.1371/journal.pcbi.1006051 (PMC5864076; doi:10.1371/journal.pcbi.1006051)

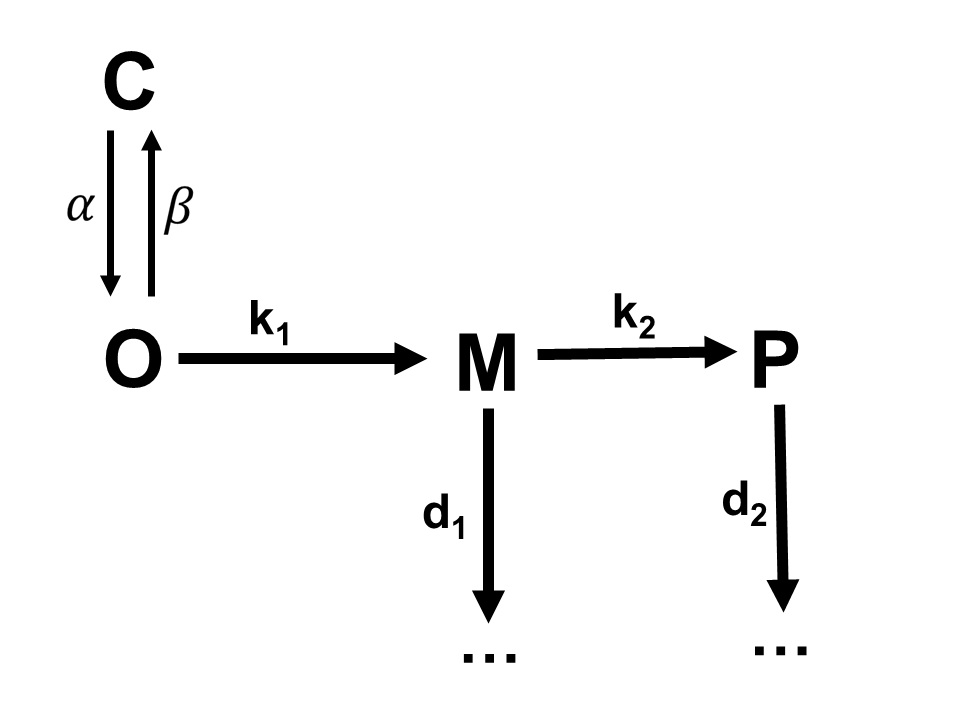

Supplement: S1 Fig — (TIF) [file pcbi.1006051.s002.tif]

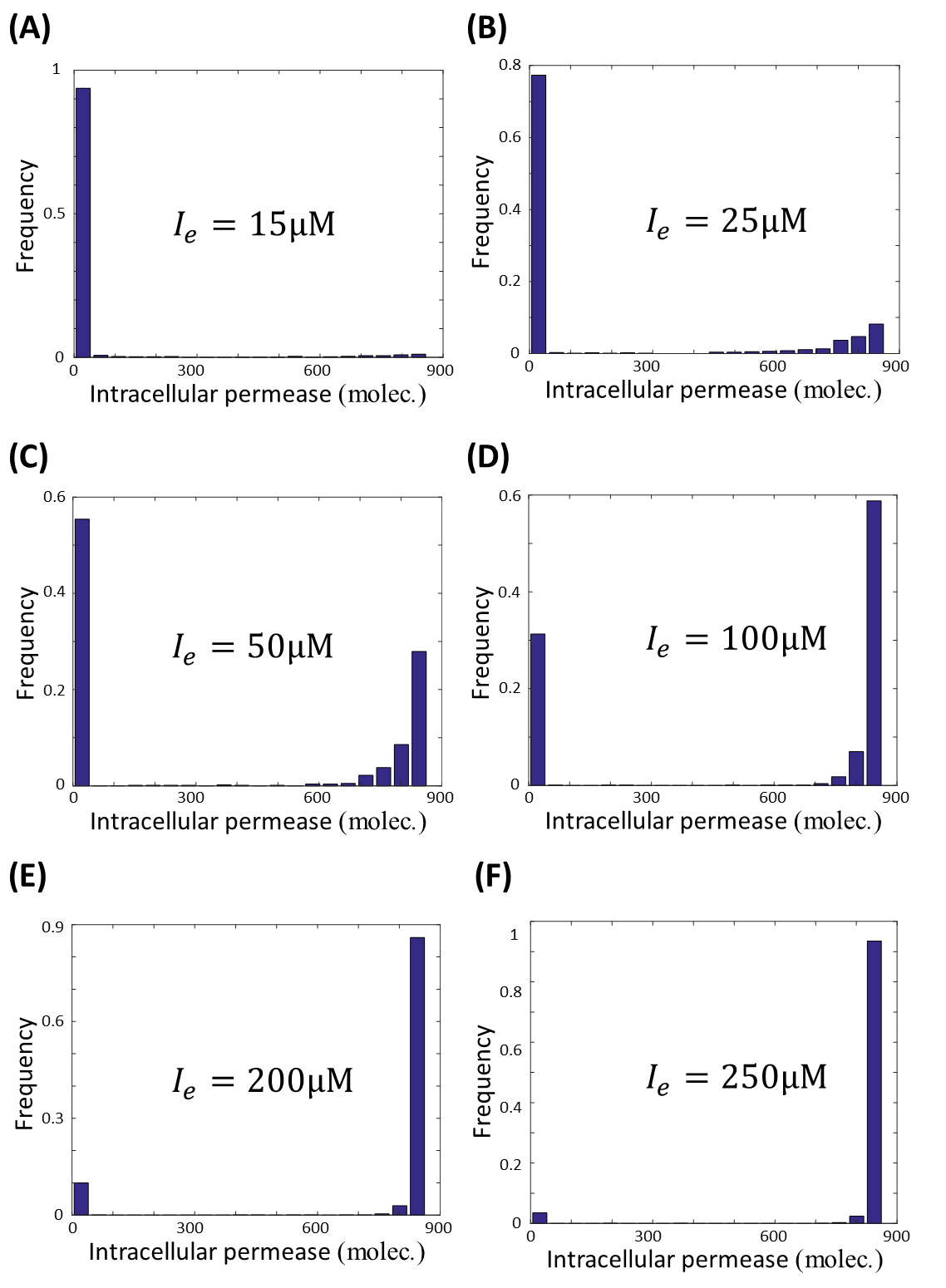

Supplement: S2 Fig — We compare the copy-number distribution of permease with different extracellular concentration of inducers Ie and show that the Ie range of the bimodal distribution is much more broader than that predicted in the deterministic bifurcation diagram(Fig 2A in the main text). (TIF) [file pcbi.1006051.s003.tif]

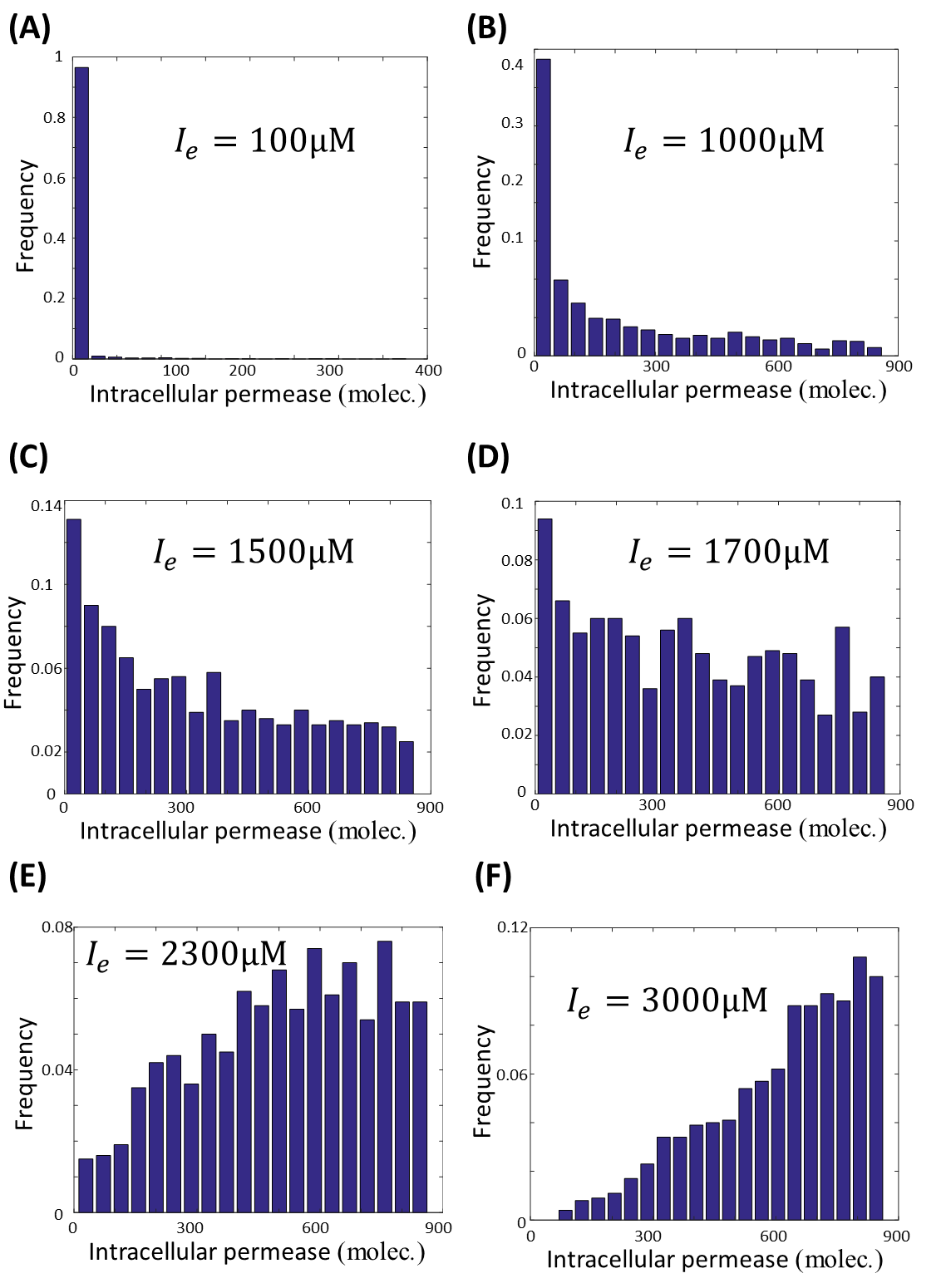

Supplement: S3 Fig — (TIF) [file pcbi.1006051.s004.tif]

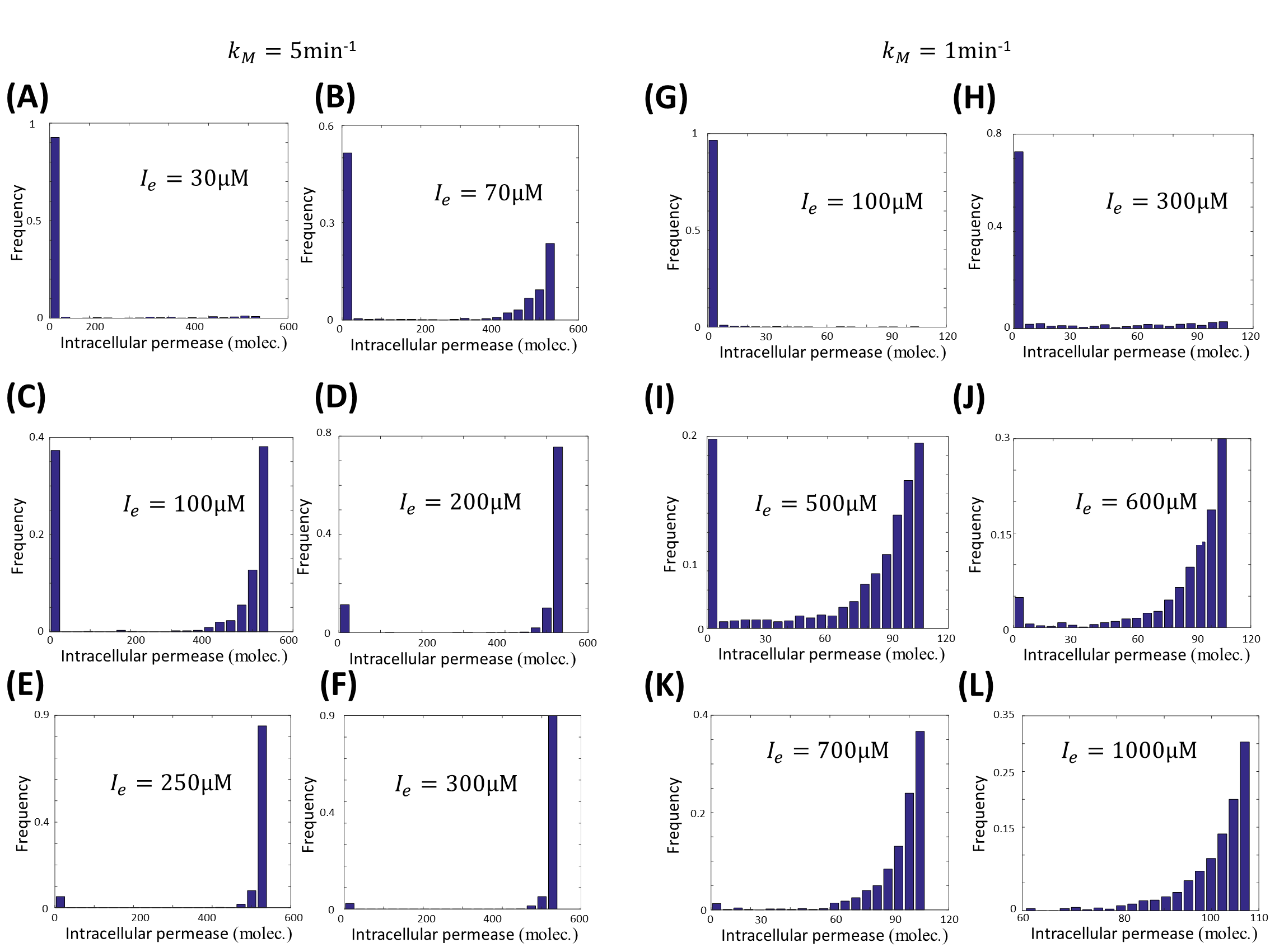

Supplement: S4 Fig — (TIF) [file pcbi.1006051.s005.tif]

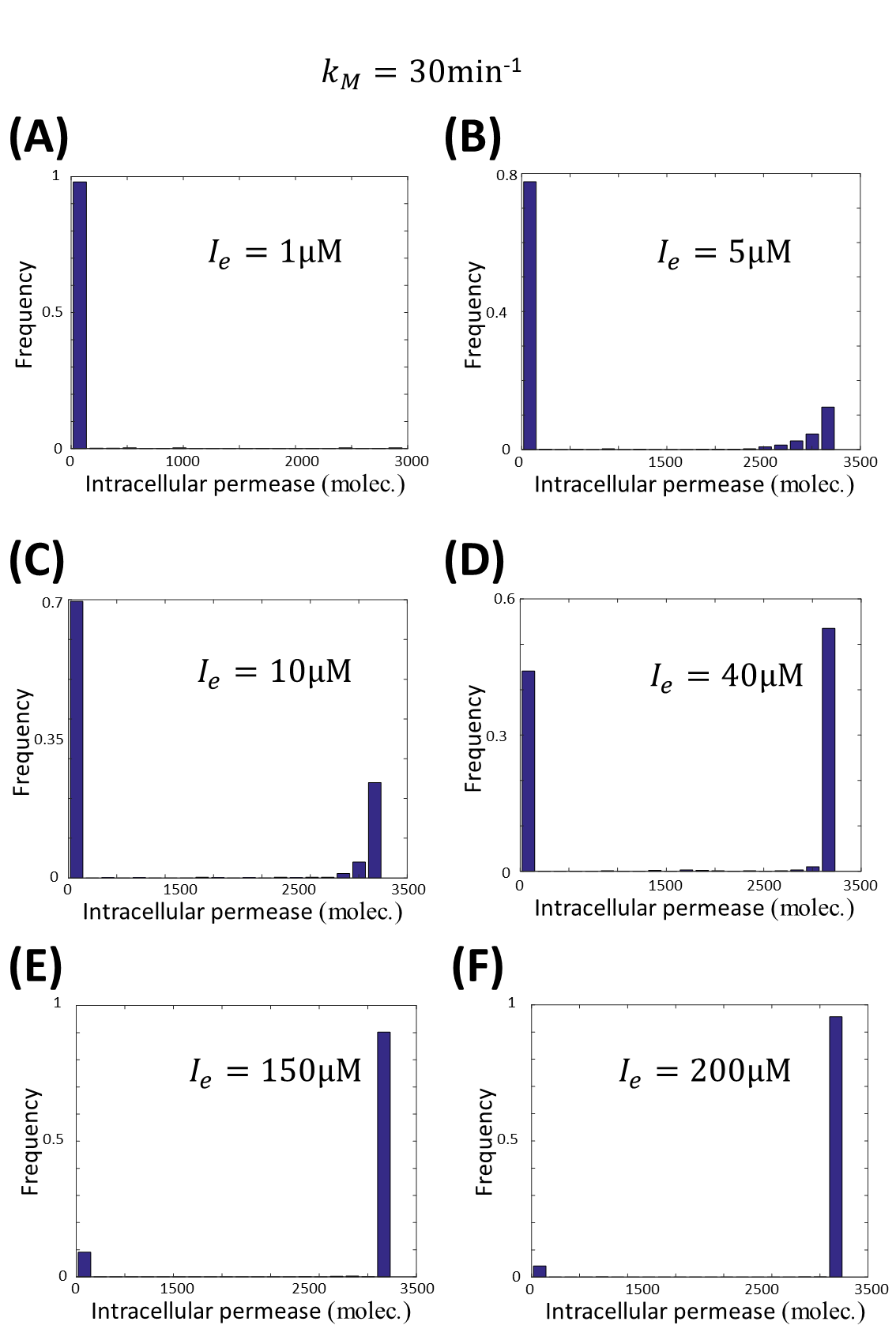

Supplement: S5 Fig — (TIF) [file pcbi.1006051.s006.tif]

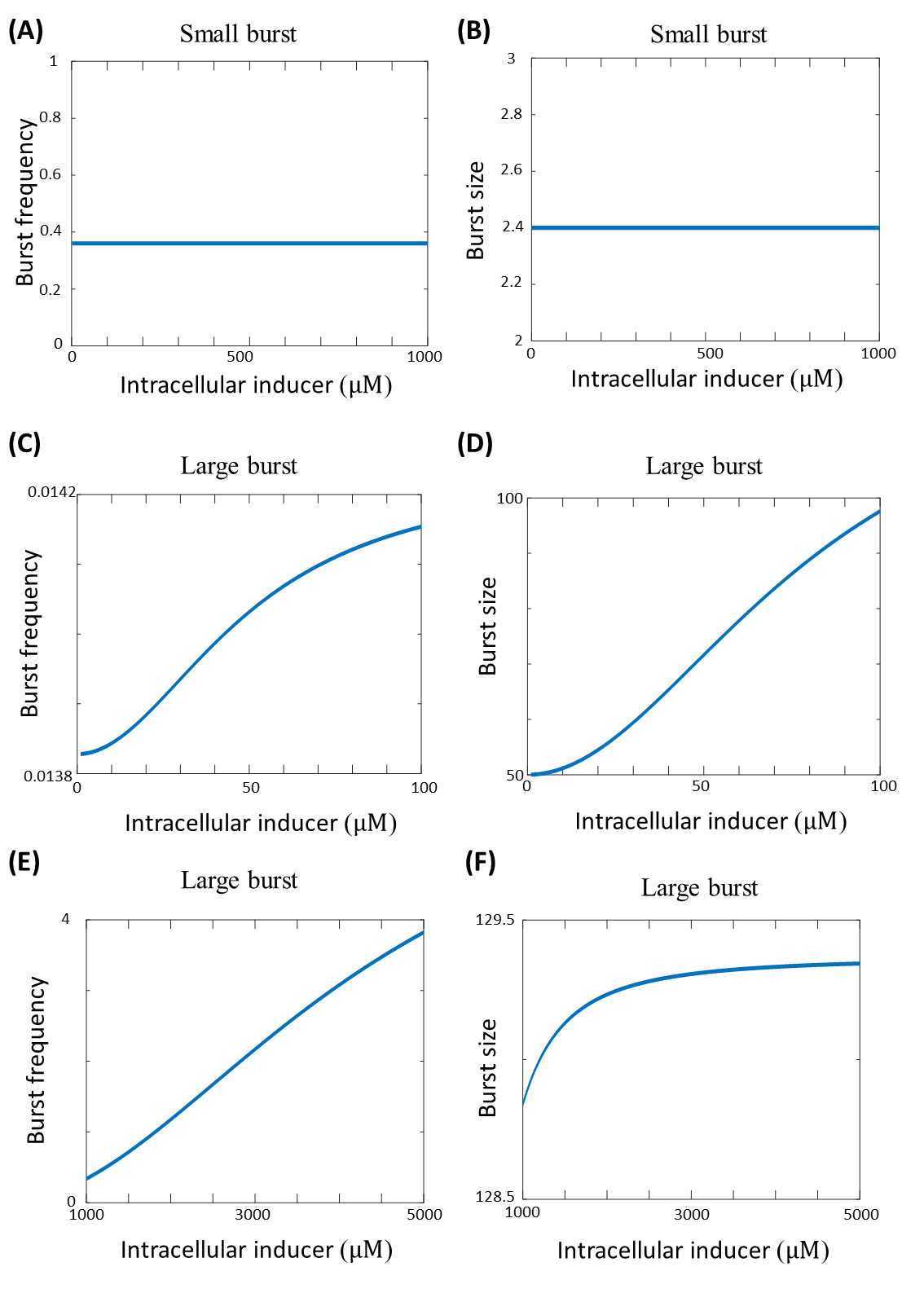

Supplement: S6 Fig — (TIF) [file pcbi.1006051.s007.tif]

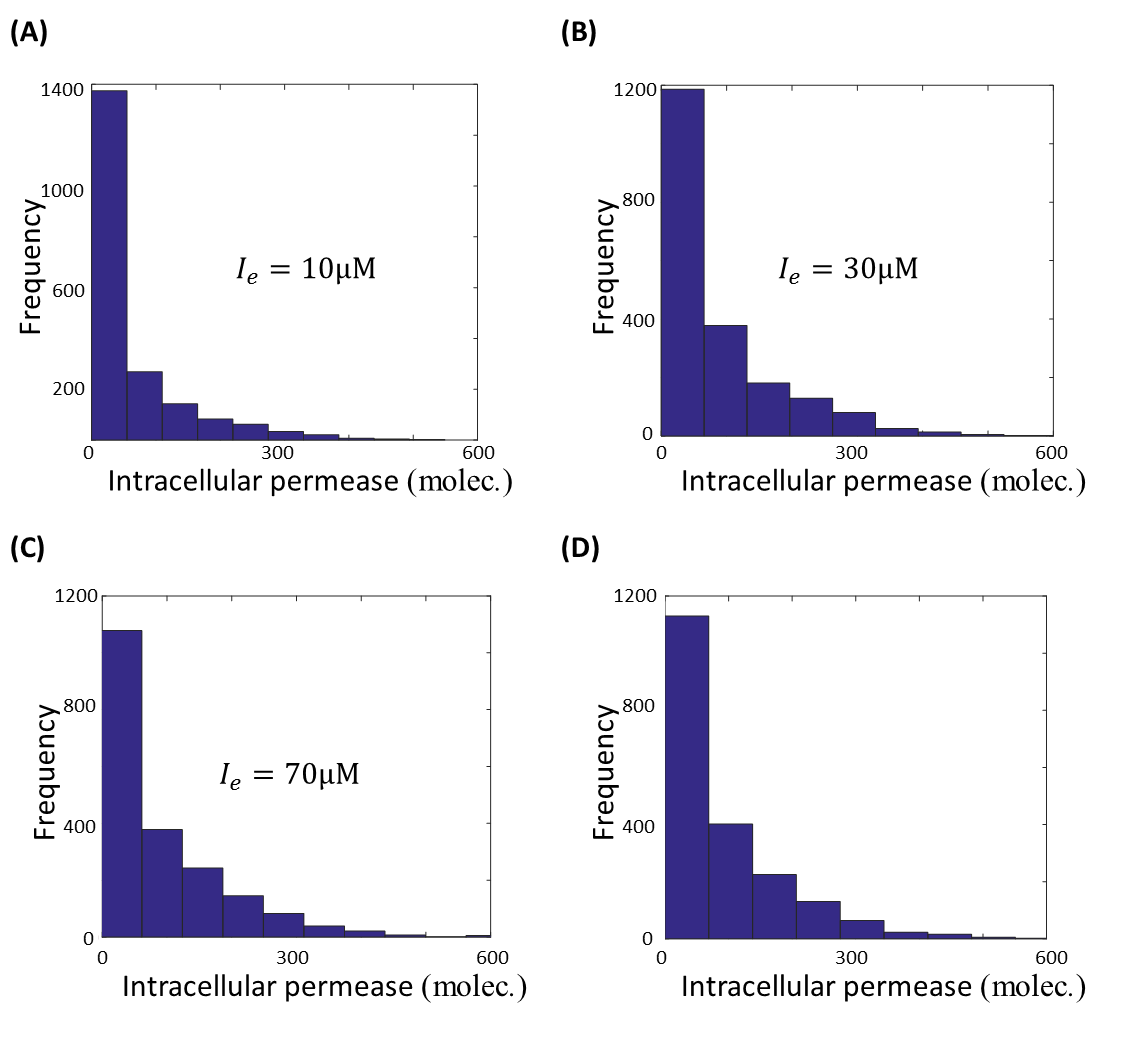

Supplement: S7 Fig — (TIF) [file pcbi.1006051.s008.tif]

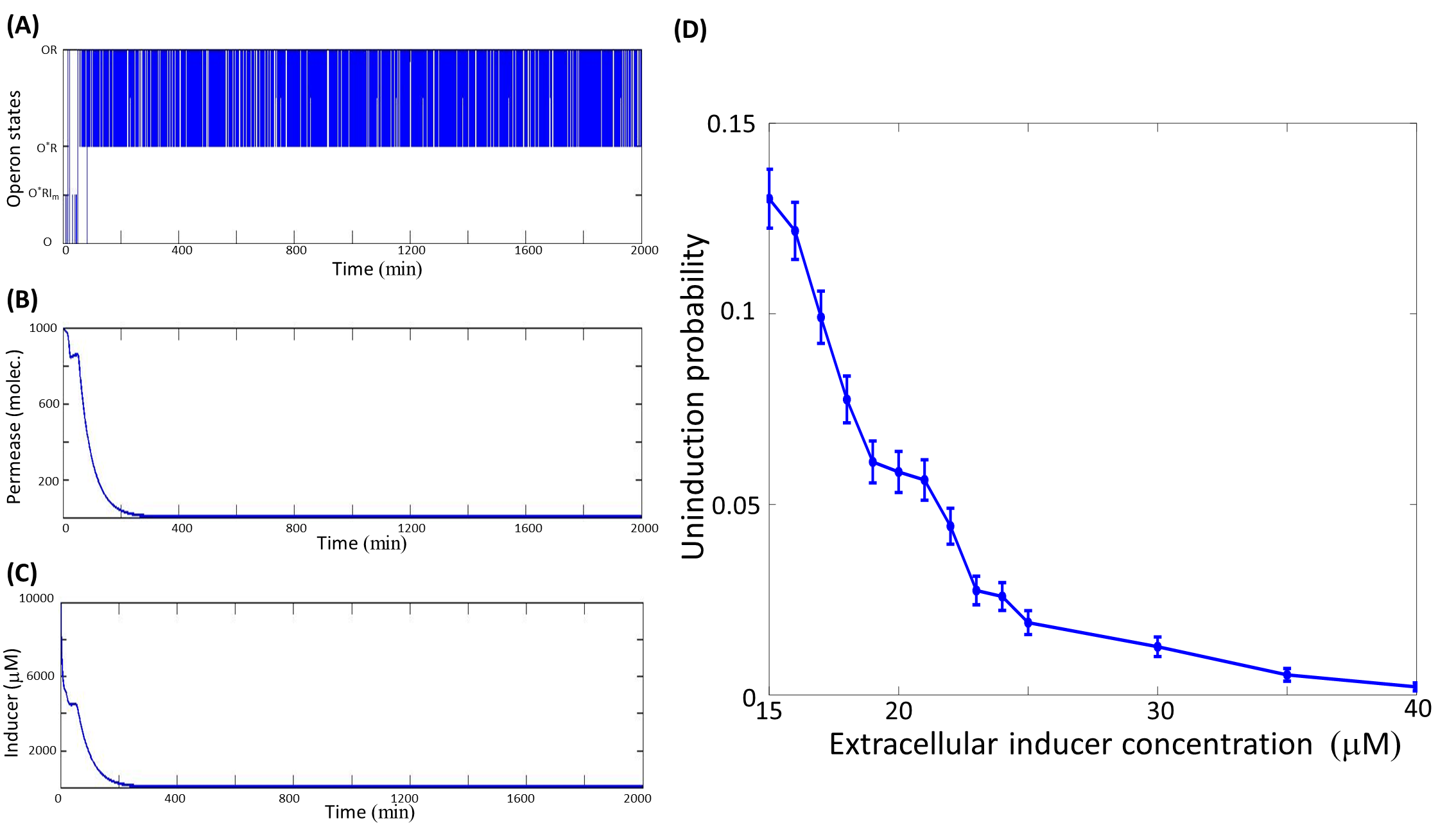

Supplement: S8 Fig — The uninduction probability nearly vanishes when the extracellular inducer concentration is only slightly larger than about 40μM. (TIF) [file pcbi.1006051.s009.tif]

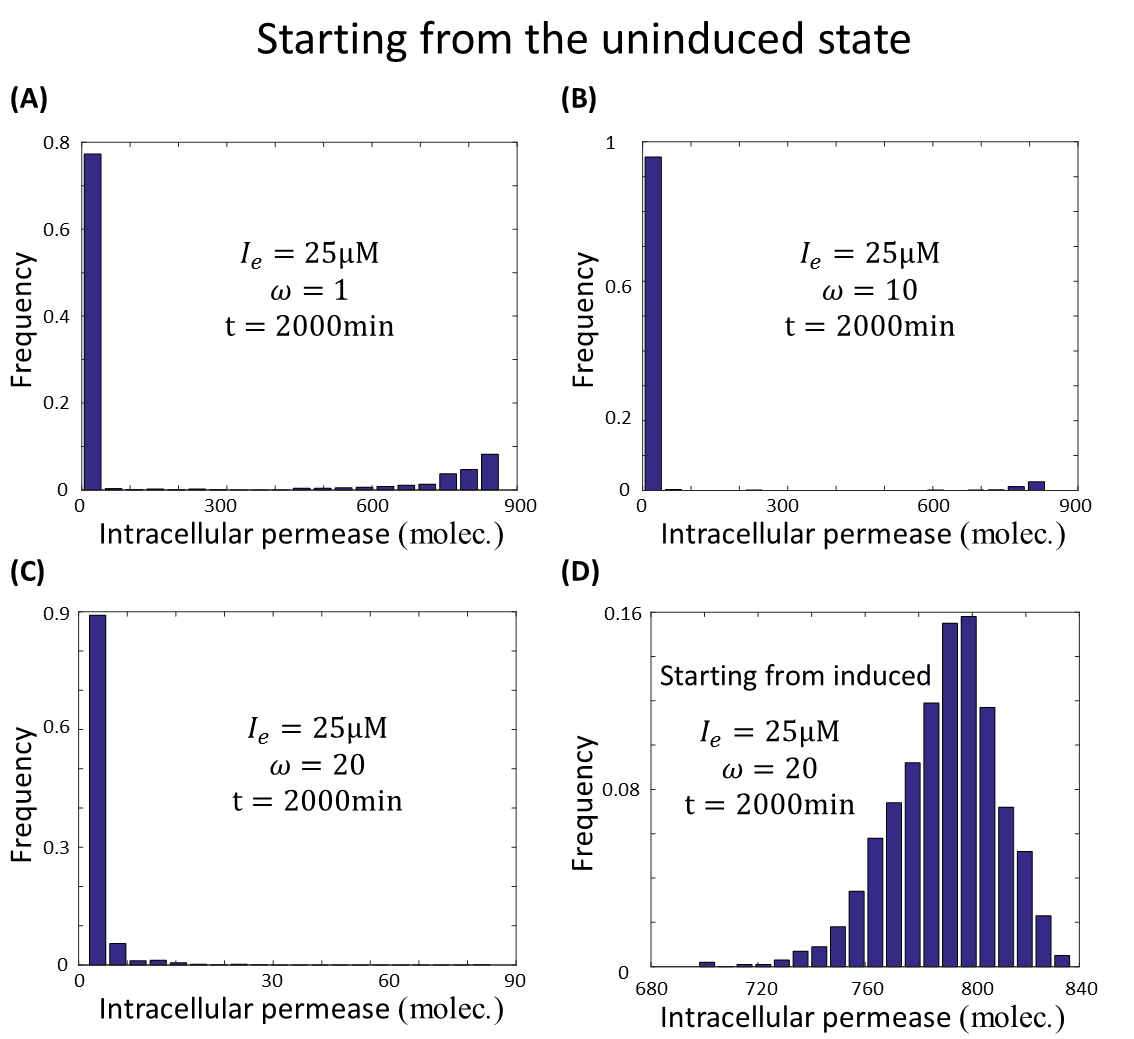

Supplement: S9 Fig — (TIF) [file pcbi.1006051.s010.tif]

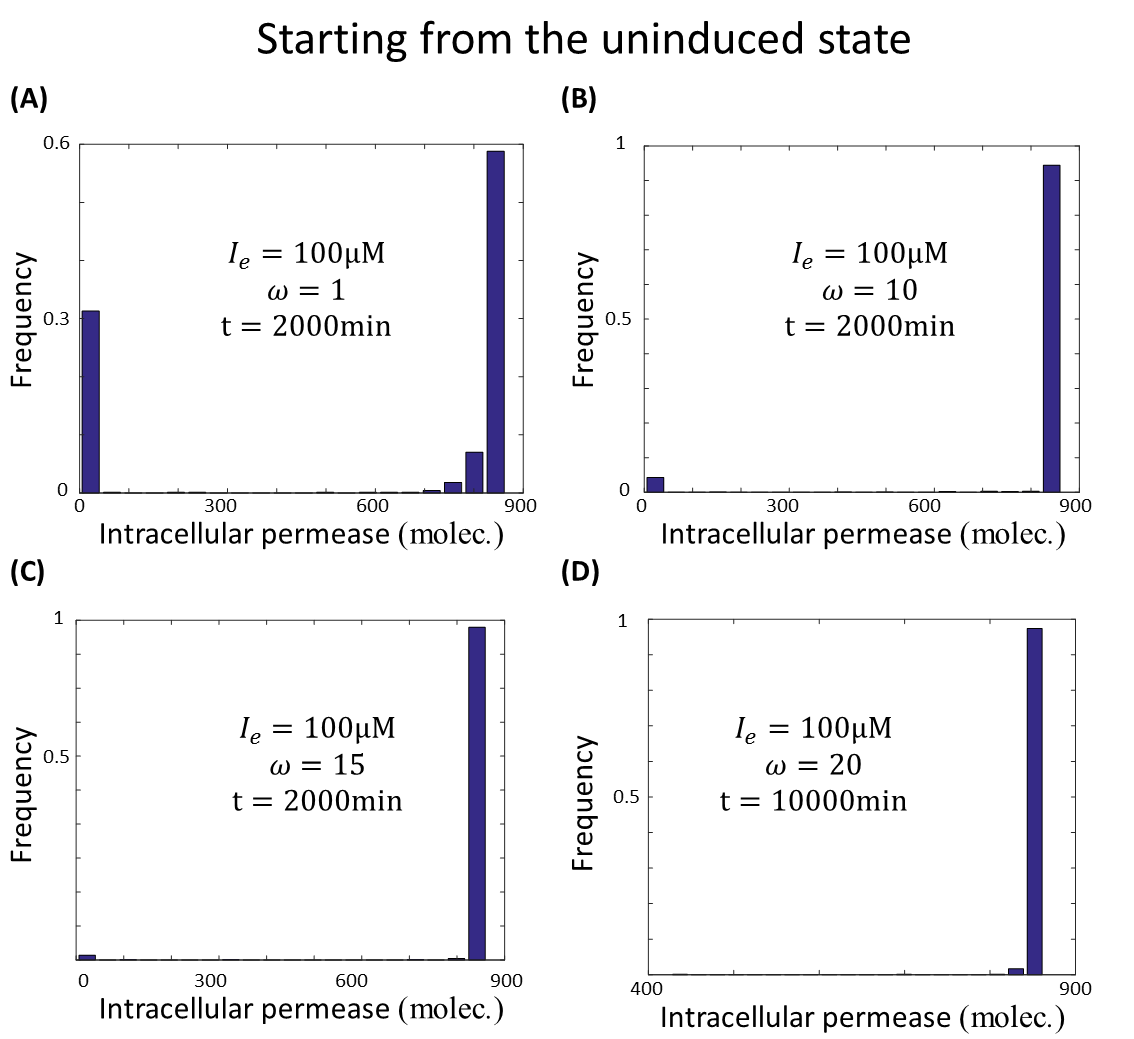

Supplement: S10 Fig — (TIF) [file pcbi.1006051.s011.tif]

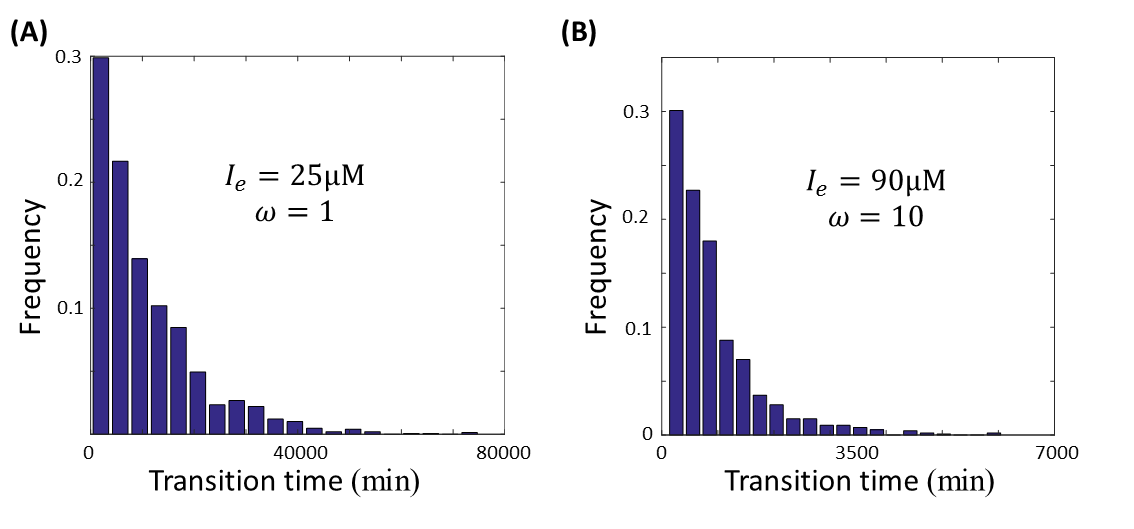

Supplement: S11 Fig — (TIF) [file pcbi.1006051.s012.tif]

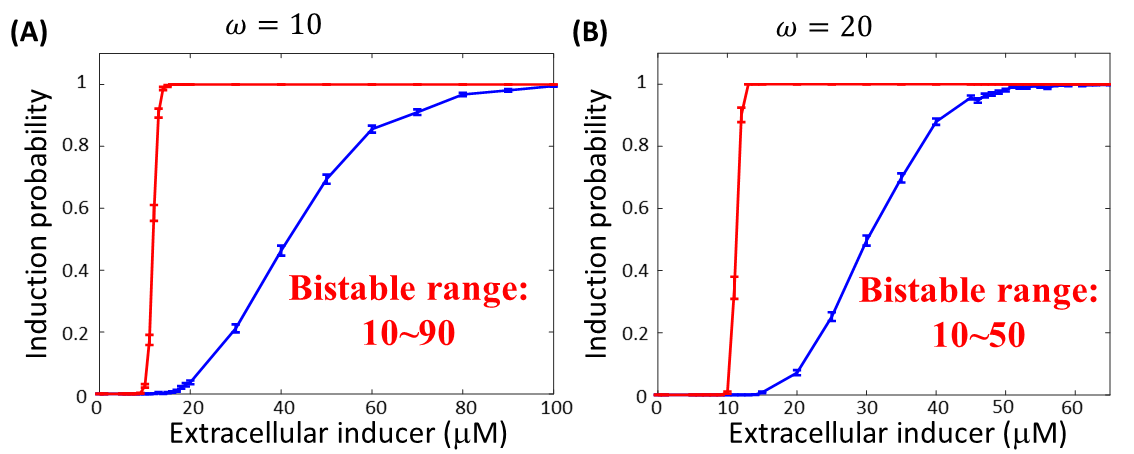

Supplement: S12 Fig — Initial conditions: uninduced (blue line) or fully induced (red line) cells with a period of T = 2000 min. (TIF) [file pcbi.1006051.s013.tif]

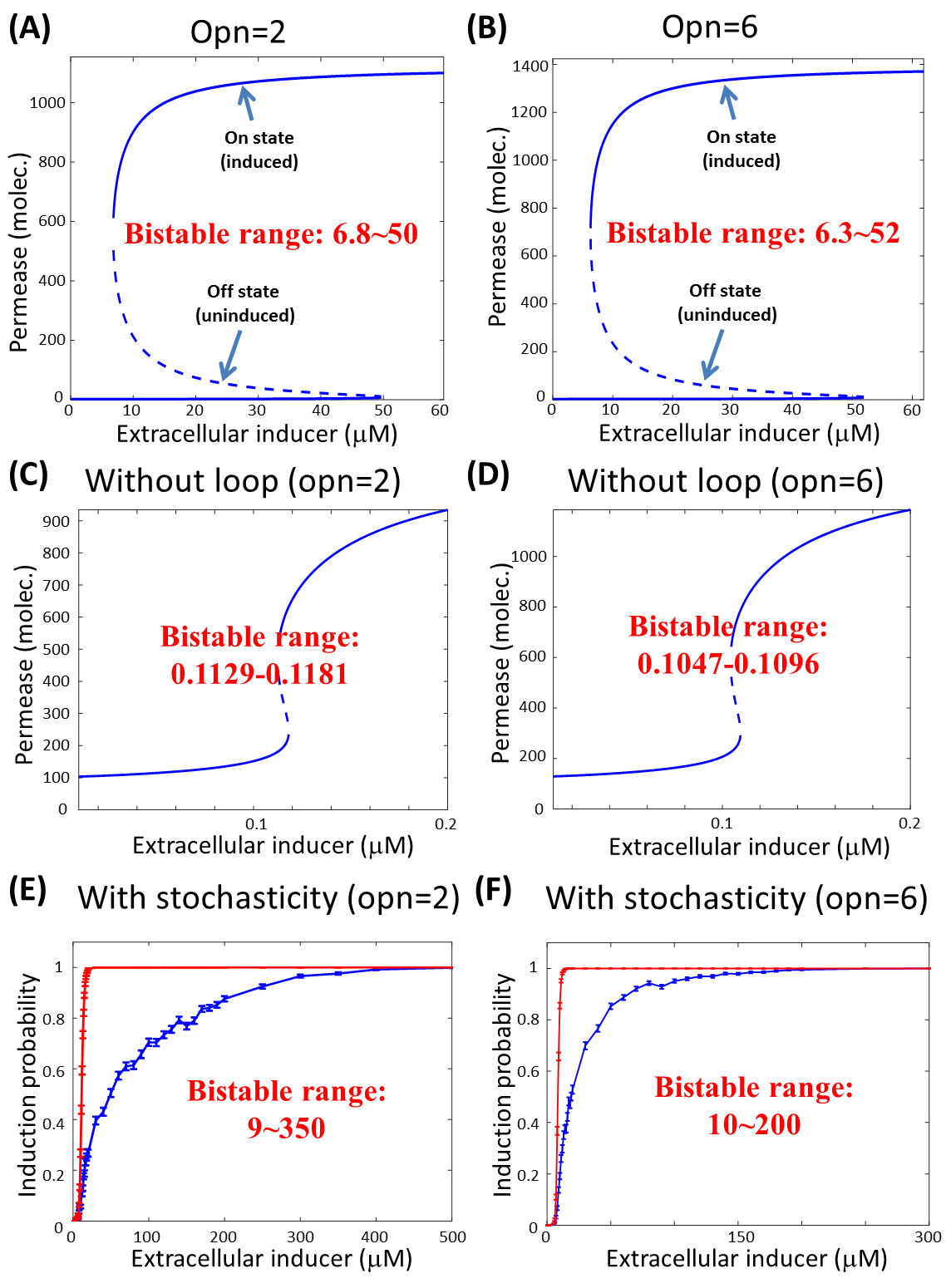

Supplement: S13 Fig — (A)(B) Deterministic bifurcation diagram for wild-type cells in which the number of operons is 2 or 6. (C)(D) Deterministic bifurcation diagrams for the repressor bound to the operon in the absence of a DNA loop with association constant that equals 5 molec.−1. (E) (F) Stochastic hysteresis response of the probability of induction. (TIF) [file pcbi.1006051.s014.tif]

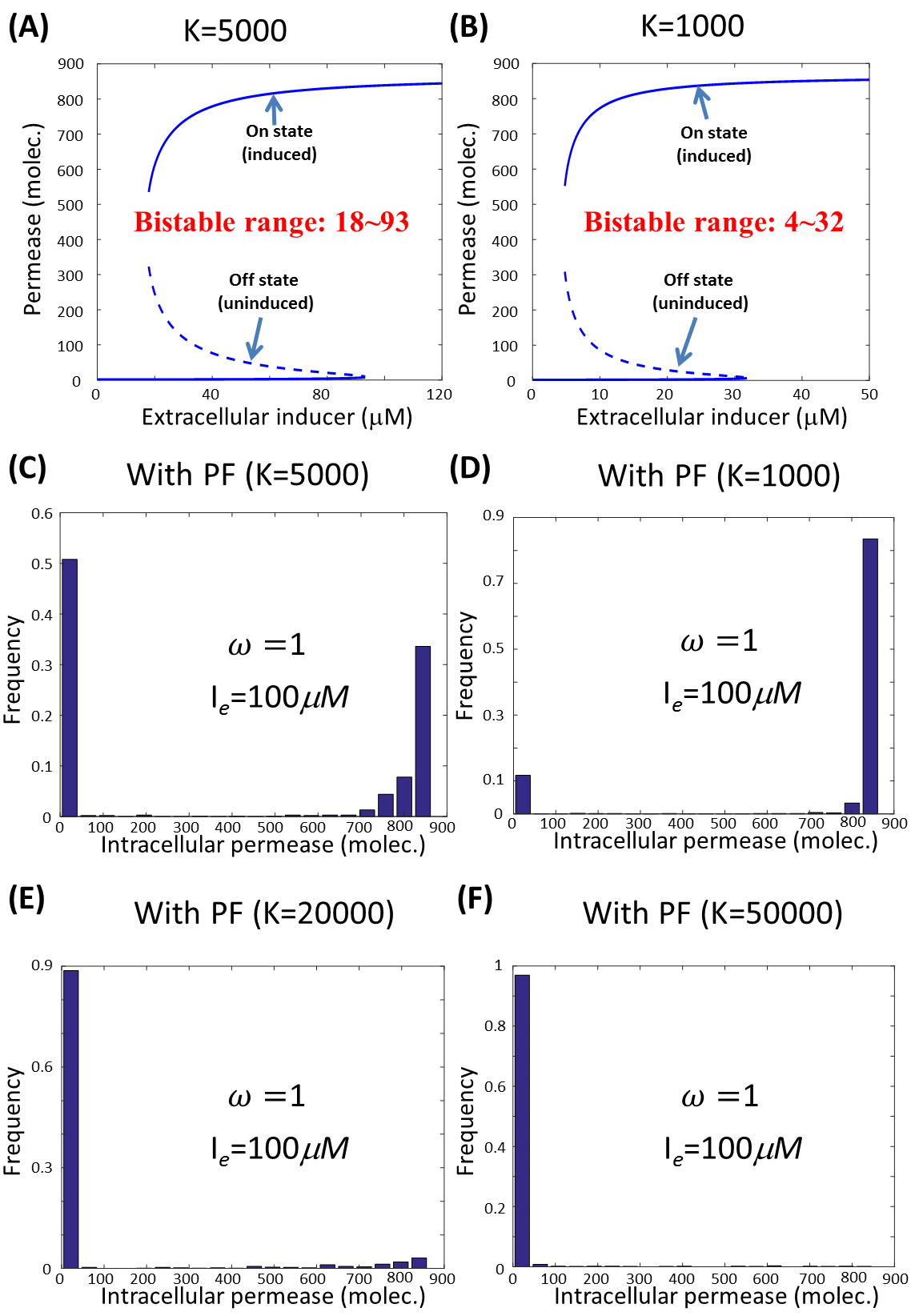

Supplement: S14 Fig — (A)(B) Deterministic bifurcation diagram tuning the strength of positive feedback. (C-F) Stationary distributions when tuning the strength of positive feedback. (TIF) [file pcbi.1006051.s015.tif]

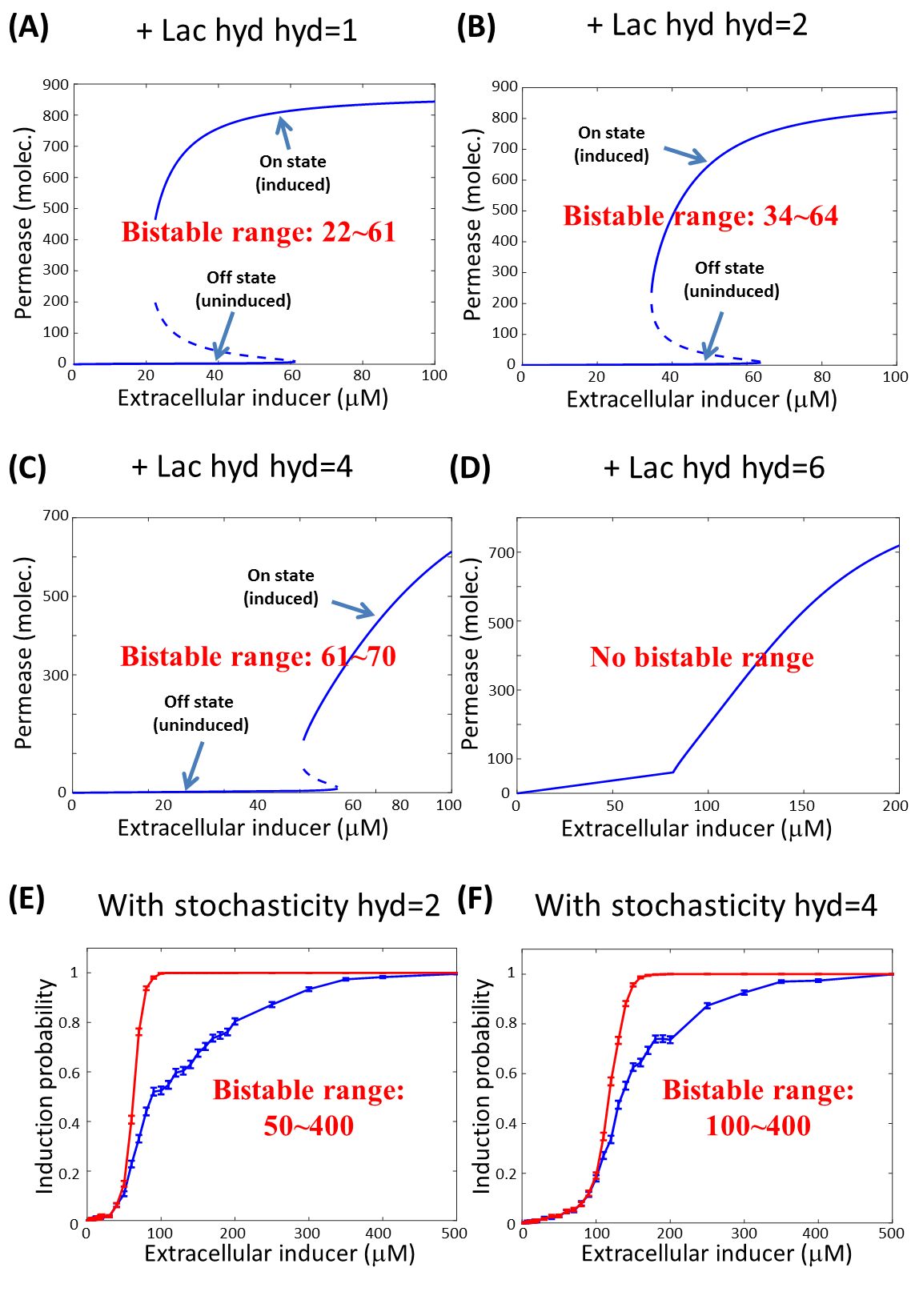

Supplement: S15 Fig — (A-D) Deterministic bifurcation diagram in which the dynamics of inducer is replaced by that of lactose. (E) (F) Stochastic hysteresis response of the probability of induction. (TIF) [file pcbi.1006051.s016.tif]
