# Supplementary material for: Relatively slow stochastic gene-state switching in the presence of positive feedback significantly broadens the region of bimodality through stabilizing the uninduced phenotypic state
Source: PLoS Comput Biol. 2018 Mar 12;14(3):e1006051. doi: 10.1371/journal.pcbi.1006051 (PMC5864076; doi:10.1371/journal.pcbi.1006051)
Supplement: S1 Table — (PDF) [file pcbi.1006051.s017.pdf]

$c=0.23\mathrm{min}^{-1}$	$k_M=8\mathrm{min}^{-1}$	$\gamma_M=0.756\mathrm{min}^{-1}$
$k_Y=1.8\mathrm{min}^{-1}$	$\gamma_Y=0.022\mathrm{min}^{-1}$	
$R_T=2\mathrm{molec.}$	$K=2500\mu M^2$	$f=1$
$r_1=0.2\mathrm{molec.}^{-1}\mathrm{min}^{-1}$	$r_{-1}=0.32\mathrm{min}^{-1}$	$K_1=0.625\mathrm{molec.}^{-1}$
$r_2=1000\mathrm{min}^{-1}$	$r_{-2}=1\mathrm{min}^{-1}$	$K_2=1000$
$r_3=8\times10^{-6}\mu M^{-2}\mathrm{min}^{-1}$	$r_{-3}=1\mathrm{min}^{-1}$	$K_3=8\times10^{-6}\mu M^{-2}$
$r_4=6\mathrm{min}^{-1}$	$r_{-4}=0.075\mathrm{molec.}^{-1}\mathrm{min}^{-1}$	$K_4=80\mathrm{molec.}$
$\gamma_I=0.012\mathrm{min}^{-1}$	$k_I=0.25\mathrm{min}^{-1}$	
